# Evaluation of Malaria Parasite Transmission Competency Using a Nanozyme-Based Immunodiagnostic Targeting Female Gamete Antigen Release

**DOI:** 10.1021/acsnano.5c10298

**Published:** 2025-10-06

**Authors:** Tabasom Haghighi, Adrian Najer, Marta Broto, Farah A. Dahalan, Alisje Churchyard, Sabrina Yahiya, Mufuliat T. Famodimu, Mark Tunnicliff, Aida Abdelwahed, Mayumi Tachibana, Tomoko Ishino, Yaw Aniweh, Gordon A. Awandare, Almahamoudou Mahamar, Leen N. Vanheer, Teun Bousema, Chris Drakeley, Alassane Dicko, William Stone, Jake Baum, Molly M. Stevens

**Affiliations:** † Department of Materials, Department of Bioengineering, and Institute of Biomedical Engineering, 4615 Imperial College London, London SW7 2AZ, U.K.; ‡ Kavli Institute for Nanoscience Discovery, Department of Physiology, Anatomy and Genetics, Department of Engineering Science, 6396 University of Oxford, OX1 3QU Oxford, U.K.; § Department of Life Sciences, Imperial College London, London SW7 2AZ, U.K.; ∥ Division of Molecular Parasitology, Proteo-science Center, 12760 Ehime University, 454 Shitsukawa, Toon, Ehime 791-0295, Japan; ⊥ Department of Parasitology and Tropical Medicine, 13290 Graduate School of Medical and Dental Sciences, Institute of Science Tokyo, 1-5-45, Yushima, Bunkyo-ku, Tokyo 113-8519, Japan; # West African Centre for Cell Biology of Infectious Pathogens (WACCBIP), College of Basic and Applied Sciences, University of Ghana, LG 54 Legon, Ghana; ∇ Clinical Research Unit of Bougouni-Ouelessebougou, Malaria Research and Training Centre, 225803 University of Sciences, Techniques, and Technologies of Bamako, Bamako BP 1805, Mali; ○ Department of Infection Biology, 4906 London School of Hygiene and Tropical Medicine, London WC1E 7HT, U.K.; ◆ Radboud Institute for Health Sciences, Radboud University Medical Centre, Geert Grooteplein Zuid 28, 6525GA Nijmegen, Netherlands; ¶ School of Biomedical Sciences, 7800 University of New South Wales, NSW 2052, Sydney, Australia

**Keywords:** nanozyme, diagnostic, malaria, Plasmodium falciparum, transmission

## Abstract

Preventing malaria parasite transmission to the mosquito vector is a key elimination challenge that could benefit from a diagnostic test that can identify transmission-competent individuals. Only sexual parasite forms, called gametocytes, which activate into gametes (gametogenesis) upon mosquito uptake or blood sampling are responsible for transmission. Herein, we devised a nanozyme-based immunoassay to detect the *Plasmodium falciparum* female gametocyte activation antigen *Pf*G377, which is released during gametogenesis. Initial validation of our nanozyme assay with cultured parasites demonstrated that levels of *Pf*G377 were higher in supernatants of activated versus nonactivated cultures and those treated with transmission blocking drugs. To define the field potential of this approach, patient samples from a clinical transmission-focused trial were used, including those receiving treatment with the gametocytocidal drug primaquine (PQ) that sterilizes gametocytes and blocks transmission. PQ-treated patient samples showed significantly lower signals of *Pf*G377 2 days after treatment, consistent with an inability of PQ-treated gametocytes to activate and release antigen upon blood sampling. This study serves as a pathfinder for field transmission rapid diagnostics to detect transmission-competent individuals, which could help revive malaria elimination strategies.

## Graphical abstract

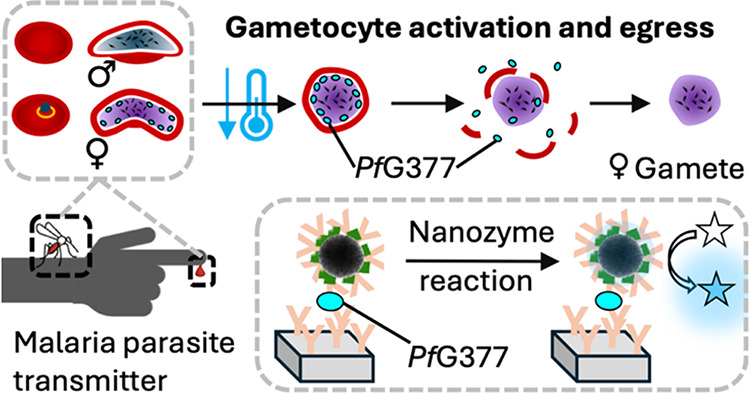

Malaria is responsible for more mortality and morbidity than any other parasitic disease in humans. In recent years, efforts to combat malaria have stagnated, recording 263 million cases and 597,000 deaths in 2023.^1^ Having a malaria-free world has been one of the main goals of the WHO and the global malaria community for many years.^2^ Given that transmission between humans and mosquitoes is essential for malaria parasite spread, local elimination and global eradication are achievable if countries successfully block the parasite two-host life cycle by interrupting it at any life cycle stage. However, while simple in concept, this task has proven challenging not least because of the difficulty of identifying asymptomatic parasite carriers that contribute significantly to the onward transmission to mosquitoes^3–6^ and the inaccessibility of a specific transmission sensor.

Transmission to the mosquito is limited to the sexual stages of *Plasmodium falciparum*, called gametocytes. Gametocytes are relatively dormant metabolically in the bloodstream. Following a female mosquito bite, however, mature stage V gametocytes in the midgut rapidly activate (within 20 min),^7^ shedding their red blood cell (RBC) coat to form gametes (gametogenesis), which mediate fertilization and continuation of the parasite life cycle in the mosquito. Gametogenesis is a well-orchestrated cellular process involving several classes of transmission-specific proteins, including those residing within osmiophilic bodies (OB). *Pf*G377 is a hydrophilic OB protein specific to female gametocytes that is expressed from stage III onward, at the onset of OB biogenesis.^8^ *Pf*G377 is essential for OB development and it is present at high concentrations.^9–11^ During activation in the mosquito, OBs migrate to the parasite plasma membrane and disappear within a few minutes, releasing their contents, including *Pf*G377, into the parasitophorous vacuole (PV).^12^ Upon subsequent rupture of the RBC membrane, *Pf*G377 is expelled into the plasma. Gametocyte activation *in vitro*, mimicking conditions within the mosquito midgut environment, identifies *Pf*G377 as one of the major proteins released into the supernatant,^13,14^ making it a potential marker for transmission-competent mature gametocytes.

To date, assays of transmissibility have mostly been based on mosquito feeding assays that provide definitive proof of gametocyte viability but are restricted to specialized laboratories with insectaries and feeding facilities and thereby costly and not scalable. An alternative approach would be to predict transmissibility depending on the presence of gametocytes. Among the available techniques to detect gametocytes, standard microscopy analysis of blood smears remains widely adopted, despite the associated low sensitivity. Alternatively, highly sensitive and selective molecular diagnostic tests including RT-qPCR^15–18^ that detect parasite RNA can quantify gametocytes or sexually committed ring-stage parasites. Contemporary point-of-care (PoC) adaptable molecular techniques, such as loop-mediated isothermal amplification (LAMP)-based assays^19^ and CRISPR-based diagnostics,^20–22^ have also been employed for species identification but are yet to be widely tested for gametocyte detection.^23^ All these molecular assays can enable measurement of the density of gametocytes in the blood of human hosts as a surrogate marker for transmission potential to mosquitoes.^24^ However, the presence of gametocytes is not a direct predictor of their transmission, especially after drug treatment. For example, low-dose primaquine (PQ) in combination with other antimalarials effectively blocks transmission 2 days after treatment, but the number of circulating gametocytes remains high despite them being inactive.^25^ Thus, measurements that solely detect the presence of gametocytes would likely result in false positives for transmission potential when using microscopy or even RNA-based methods.

Immunoassay-based rapid diagnostic tests (RDTs) that use finger-prick blood samples to detect malaria disease are the current gold standard.^1^ Therefore, a similar transmission-specific RDT might be more easily implemented compared to more technically demanding diagnostic tests. In terms of immunoassay development specifically for transmission stages, previous attempts include detection of antibodies^26^ and antigens.^27^ The latter is a saliva-based test that measures the female gametocyte-specific protein PSSP17 released by parasites in the human host,^27^ but it remains to be seen whether this test offers the necessary specificity.^28^ Whether PSSP17 persists after drug treatment would need to be evaluated too, since relying on an antigen secreted in the human bloodstream and transported to saliva might be problematic when studying drug action, as is the case when measuring presence of histidine rich protein-2 (HRP2, the main target of current malaria disease RDTs)^29^ or gametocyte RNA.^25^ Developing RDTs based on alternative biomarkers, including those specific for transmissible parasites, could facilitate not just carrier detection but also efforts to break the malaria transmission cycle. A highly sensitive diagnostic test that can detect the transmission potential of all parasite carriers within the population, including asymptomatic individuals,^30^ would allow for targeted administration of transmission blocking drugs such as PQ, methylene blue (MB),^31–33^ or compounds currently under development,^34,35^ to individuals or groups that contribute most to local transmission, for example, as part of a focal screen and treat program. Thus, detection of mature and activatable transmission stage carriers through a specific transmission test could justify the use of combination drug regimens also in apparently healthy, asymptomatic individuals.

Here, we propose an assay to predict malaria transmission competency that detects the presence of mature, activatable stage V gametocytes in human blood samples by measuring *Pf*G377 released during gametogenesis after sampling. We have developed a nanozyme-linked immunosorbent assay (NLISA) platform for *Pf*G377 detection using our previously designed platinum nanocatalysts (PtNCs)^36–39^ as the assay signal generating moiety (Figure 1). We first evaluated our *Pf*G377 NLISA using recombinant antigen before testing samples from cultured *P. falciparum* gametocytes under various conditions, including after drug treatment. We then demonstrated the performance of our NLISA using patient samples from Ghana (malaria positive versus negative) and from a recent transmission blocking drug trial in Mali (mosquito infection after various drug treatments).^33^ This study represents a first step toward a blood-based transmission RDT to detect individuals capable of transmitting malaria, a powerful tool to guide and monitor clinical drug trials, as well as current and future elimination/eradication efforts.

**Figure 1:**
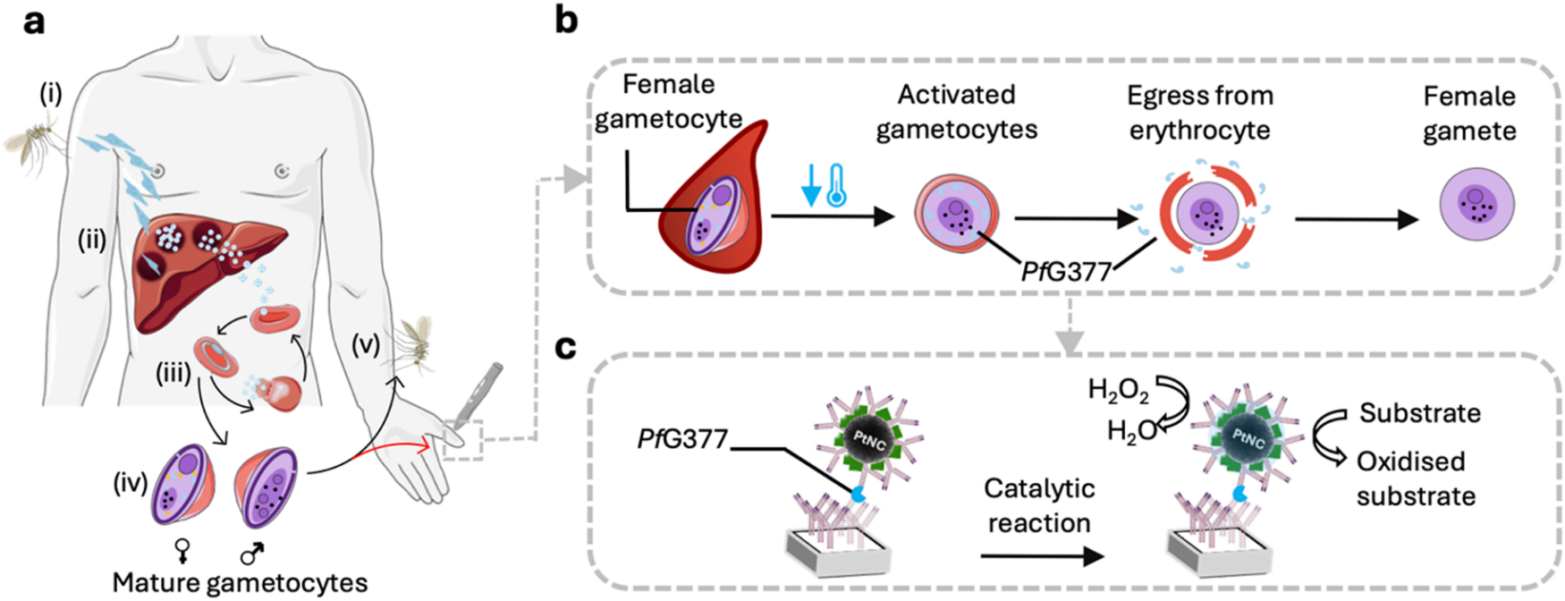
Schematic showing *Pf*G377 biomarker origin and the nanozyme-based assay design. (a) Life cycle of the malaria parasite, starting with (i) sporozoite injection by a mosquito, (ii) liver infection and transformation from sporozoite to merozoites, (iii) release from the liver and infection of red blood cells (RBCs) by merozoites followed by asexual multiplication, while (iv) 0.2–1% of asexual blood-stage parasites transition to the sexual pathway creating male and female gametocytes. These gametocytes are then taken up by the mosquito vector (v) to continue the life cycle in the invertebrate host. (b) Female gametocyte maturation journey immediately after uptake in the mosquito host. The temperature drop upon blood sampling for our experiments facilitates activation of gametocytes and formation of gametes (gametogenesis) as would happen in the mosquito vector. Upon activation, gametocytes egress from their red blood cell host membrane releasing gametes. *Pf*G377, which starts to be expressed in stage III gametocytes and is stored in OBs, is released from OBs to the PV and then into the plasma during this activation process of stage V gametocytes. (c) Catalytic nanozyme-based immunoassay setup for the detection of released *Pf*G377 for the evaluation of transmission competency using antibody-functionalized Pt nanocatalysts (PtNCs) that generate a colorimetric readout. Schematic modified from Servier Medical Art Web site CC-BY 4.0.

## Results and Discussion

### Incorporation of Platinum-Based Nanozymes in an Immunoassay for the Detection of *Pf*G377

Nanozymes, which are nanosized catalytically active materials, have recently attracted attention as a highly effective signal-generating moiety for immunoassays, while allowing implementation of the WHO REASSURED criteria for RDTs.^40,41^ We previously developed Pt nanocatalysts (PtNCs) as nanozymes for immunoassays for the sensitive PoC detection of viral antigens and various nucleic acids.^36,37^ PtNCs were prepared here using a modification of the method introduced by Loynachan et al. (Figure 2a).^36^ The 15 nm gold seeds used in this synthesis were prepared following the modified Frens’ method (Figure S1).^42^ Gold nanoparticle (AuNP) seeds were then coated with polyvinylpyrrolidone (PVP) and platinum (Pt) deposition was initiated by adding l-ascorbic acid (in excess) as a reducing agent, followed by a fast injection of chloroplatinic acid (H_2_PtCl_6_). The reaction completion was confirmed by observing a color change from red to black.

**Figure 2:**
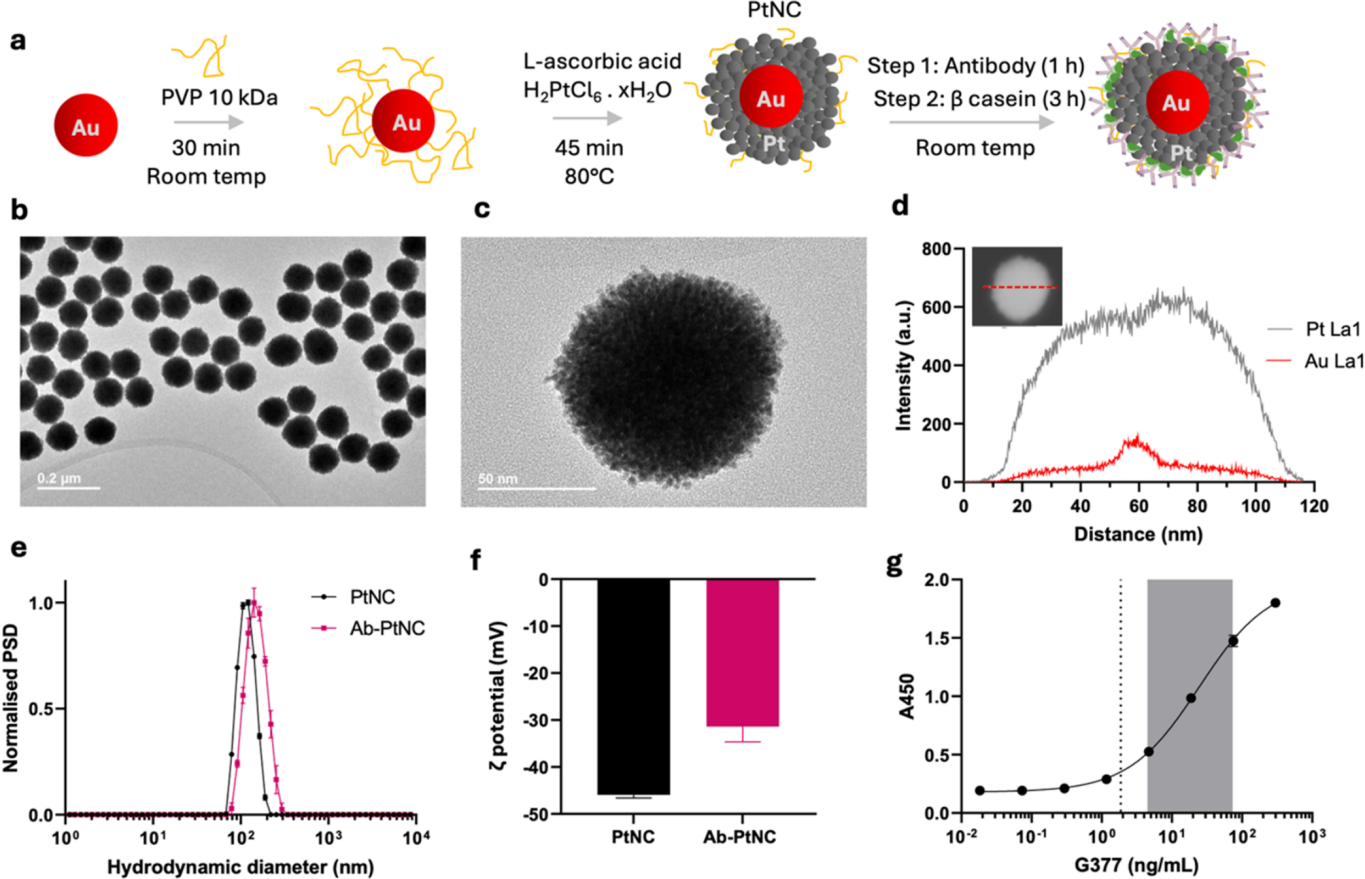
Synthesis and characterization of PtNCs for the detection of *Pf*G377. (a) Schematic showing synthesis of Au–Pt core–shell structures (PtNCs). 15 nm gold nanoparticle seeds were overgrown with platinum in the presence of l-ascorbic acid as a reducing agent and polyvinylpyrrolidone (PVP) as a stabilizer. The synthesized PtNCs are then functionalized with antibodies through physisorption. β-casein is used to block the remaining bare sites on the PtNCs to avoid nonspecific interactions with the assay matrix. Schematic modified from Servier Medical Art Web site CC-BY 4.0. (b, c) Transmission electron micrographs (TEM) of PtNCs. (d) High-angle annular dark-field STEM (HAADF-STEM) image (inset) with energy-dispersive X-ray (EDS) line scan profile recorded along the central axis of an individual 120 nm PtNC, which confirmed the core–shell structure (the full image panel can be found in Figure S2). (e, f) Characterization of the bare and antibody-functionalized PtNCs by dynamic light scattering (DLS) and zeta potential measurements. Data represents mean ± s.d. (*n* = 3). PSD, particle size distribution. (g) Nanozyme-linked immunosorbent assay (NLISA) calibration curve using a serial dilution of recombinant *Pf*G377 in phosphate-buffered saline with 0.05 wt % Tween 20 (PBST) matrix; dashed line indicates the limit of detection of the assay (2.8 ng/mL), and the gray area highlights the working range (5.7–52 ng/mL). A450, absorbance measured at 450 nm. Data represents mean ± s.d. (*n* = 3).

Transmission electron microscopy (TEM) images (Figure 2b,c) confirmed the monodispersity of the synthesized PtNCs and highlighted their porous urchin-like morphology in agreement with previous results.^36^ This morphology is critical to the catalytic activity of the particles, providing a large catalytically active surface area. Dynamic light scattering (DLS) confirmed colloidal stability as well as particle size corresponding to the TEM size and monodispersity without aggregation. Zeta potential characterization of the particles revealed values around −40 mV, which is in agreement with our previous work.^36^ High-angle annular dark-field STEM (HAADF-STEM) imaging and an energy-dispersive X-ray (EDS) line scan profile recorded along the central axis of an individual 120 nm PtNC show a significant Pt Lα1 signal intensity across the central axis of the particle and a much weaker Au Lα1 signal intensity with a small peak right in the middle of the central axis of the particle (Figure 2d; the full image panel can be found in Figure S2). This intensity profile confirms the presence of a high-density platinum shell (as indicated by the high signal intensity) around a gold nanoparticle core. The weak Lα1 signal intensity of the Au is because the Au seed is significantly shielded from the X-ray radiation by the high-density Pt shell existing on the surface of AuNPs. This low signal further confirms that Au is buried inside the nanoparticle structure, and the particles exist as a core–shell structure.

After synthesis and full characterization, PtNCs were decorated with antibodies specific to the assay target of interest, female gametocyte-specific antigen *Pf*G377. Purified rabbit anti-*Pf*G377 polyclonal antibodies^11^ were conjugated onto the PtNCs through passive physisorption. A range of specific conjugation buffer pH and antibody loading densities were tested (Figures S3–S5) and the optimized conjugation conditions of pH 7.0 and a theoretical value of 600 antibodies per PtNC were selected for subsequent experiments. The signal production in the NLISA procedure is similar to that in an ELISA, but the detection unit is the nanozyme (PtNCs). A colorimetric signal is generated after the addition of 3,3′,5,5′-tetramethylbenzidine (TMB) and hydrogen peroxide substrates to the PtNCs, followed by incubation, and finally the addition of a concentrated sulfuric acid stop solution to produce a measurable signal at 450 nm (yellow color).

To design the final sandwich NLISA platform, a checkerboard titration experiment was conducted in which the concentrations of the capture antibodies and the Ab-PtNC detection units were optimized simultaneously, and the ideal ratio between the two components was established (Figure S6). The sensitivity of the finalized NLISA platform was examined by measuring the assay signal in response to a concentration series of recombinant *Pf*G377 antigen and equated to 2.8 ng/mL of *Pf*G377 protein spiked into the PBST matrix (Figure 2g). As a control, an equivalent NLISA for HRP2 was established in parallel using commercial monoclonal anti-HRP2 antibodies on PtNCs, which revealed a limit of detection (LoD) at 0.33 ng/mL (Figure S7). Performance of the *Pf*G377 assay was also validated using gametocyte culture media, containing a large proportion of amino acids and small molecules as well as 5 vol/vol % human serum, as a more realistic assay matrix (Figure S8). Comparing the standard calibration curve of a serial dilution of *Pf*G377 spiked into gametocyte culture media (with human serum) to PBST revealed that the gametocyte media did not induce extra nonspecific interactions. The assay’s detection limit and dynamic range were unaffected by this new matrix, which further confirmed the robustness of the assay. The use of nanozymes offers several advantages, including generation of highly sensitive diagnostic tests and simple transfer to PoC setups such as lateral flow immunoassay (LFIAs).^40,41^ To verify the transferability of our *Pf*G377-NLISA to such LFIA designs, we have performed half-dipstick immunoassays using our *Pf*G377-PtNCs and CN/DAB (4-chloro-1-naphthol/3,3′-diaminobenzidine, tetrahydrochloride) with hydrogen peroxide as the amplification reagents, which revealed similar LoD compared to our *Pf*G377-NLISA (2.8 ng/mL, Figure 2g), detecting *Pf*G377 in serum at 1.3 ng/mL after amplification and using mobile phone readout (Figure S9).

### *In Vitro* Evaluation of the NLISA Platform Reveals Successful *Pf*G377 Detection upon Gametocyte Activation

When a mosquito blood-feeds from a parasite-carrying human, the parasites experience a temperature drop from 37 °C to below 30 °C,^7,43^ and are exposed to xanthurenic acid (XA),^44^ which activates mature stage V gametocytes present to rapidly form gametes within the sample. This can be accurately mimicked *in vitro* by the addition of XA to aid efficient gametocyte activation upon a temperature drop. Gametogenesis is accompanied by *Pf*G377 antigen release when the erythrocyte containing a female gametocyte ruptures, serving as a potential biomarker for transmission competency (Figure 3a). To evaluate our NLISA platform, we employed *P. falciparum* gametocytes (NF54 and NF135 strain) cultured in human serum-containing culture medium, assessing our ability to detect *Pf*G377 released into the serum upon gametocyte activation.

As a standardized control, cultures were evaluated for maturity, before and after the temperature drop and XA addition and visualized by Giemsa-stained thin blood smears (Figure 3b i-ii pre- and iii postactivation). The morphological change of gametocytes from crescent shape to spherical cells upon activation confirmed the maturity of the gametocyte cultures used. Additionally, the characteristic motile flagellar microgametes on the male activated cells (termed exflagellation centers) further confirmed the success of activation. Typical gametocyte densities for these cultures ranged from about 100–300 exflagellating male gametocytes/μL, corresponding to about 400–1200 female gametocytes/μL for the stock cultures, as the female to male gametocyte ratio is typically about four.^45^ Next, immunofluorescence imaging was performed to visualize the *Pf*G377 protein before activation to confirm the suitability of polyclonal anti-*Pf*G377 antibodies^11^ in staining OBs where this antigen resides in mature female gametocytes (Figure 3b (iv)). The *Pf*G377 antibodies consistently only labeled a subset of gametocytes in the typical dotted morphology previously identified as OBs in female stage IV–V gametocytes.^9–11^

We next used the activated and nonactivated samples to see whether *Pf*G377 can be detected after activation using our developed NLISA platform. Thirteen independent gametocyte-containing blood samples (from 13 different cultures) were prepared by mixing gametocyte culture pellets and human RBCs with human serum to reach a final 50% v/v human RBC to human serum ratio at 0.1% gametocytemia (equals a gametocyte density of about 5000 gametocytes/μL, male and female, without assessing maturity, in the final mix). This composition mimics patient blood samples, and these were kept at either 37 °C throughout the experiment to prevent gametocyte activation or they were activated for 1 h by allowing them to cool down to room temperature. After 1 h, both the activated and nonactivated (control) samples were centrifuged at 37 °C and the supernatant was analyzed on the *Pf*G377-NLISA platform. Statistically significantly higher amounts of *Pf*G377 were released from the activated gametocyte-containing samples versus the control samples kept at body temperature (Figure 3c). The signal observed for the nonactivated samples, kept at 37 °C, was only slightly higher than that of the assay background. Supernatants from asexual cultures revealed no signal for *Pf*G377 with our NLISA, while the equivalent HRP2-NLISA showed the expected signal, confirming the specificity of the *Pf*G377-NLISA toward sexual stages (Figure S10, supernatants from 1% asexual late-stage overnight cultures, 3D7 strain, yielding ca. 5% ring-stages at harvesting stage). These results suggest that cooling down the gametocyte samples, which triggers activation, causes release of *Pf*G377 into the rupturing RBC and subsequently, the gamete’s environment, which was then detected using our *Pf*G377-NLISA. This observation is in agreement with previous mass spectrometry studies that have identified G377 as a major antigen appearing in the supernatant after gametocyte activation.^13,14^

In order to assess the LoD of our assay, in terms of female gametocytes/μL, serial dilutions of gametocyte culture in gametocyte medium were prepared at 37 °C. Dilutions were cooled down to room temperature for 1 h to allow for complete activation, and the resulting *Pf*G377 signal was measured on the NLISA platform. In this experiment, the exflagellation density of the culture was used as an indicator for gametocyte activation success and a surrogate marker for estimating the number of female gametocytes present in the culture (multiply the number of exflagellation centers (males)/μL by 4 to yield female gametes/μL). In natural *P. falciparum* infections, the sex ratio of the gametocyte population is typically female-biased with 3 to 4 females per 1 male gametocyte.^45^ Cultured gametocytes successfully maintain this sex ratio.^46^ It was found that the assay could detect down to approximately 51 female gametocytes/μL of a gametocyte sample (Figure S11). Although very low gametocyte numbers below our LoD can cause mosquito infection, higher gametocyte numbers above our LoD are likely more important for transmission due to an increase in oocyst numbers in mosquitoes with increasing gametocyte density.^47^ Importantly, our assay was designed to distinguish viable from nonviable gametocytes, which is key for successful transmission, and it is also relevant when detecting symptomatic parasite carriers. Use of monoclonal anti-G377 antibodies may facilitate future improvements to reduce the LoD, aligning with NLISA detection of viral antigens down to an LoD of about 0.8 pg/mL when using high-affinity binders in the same setup^36^ (compared to *Pf*G377 here 2.8 ng/mL LoD, Figure 2g). The current setup was nonetheless deemed sufficient for the subsequent proof-of-concept trials.

**Figure 3:**
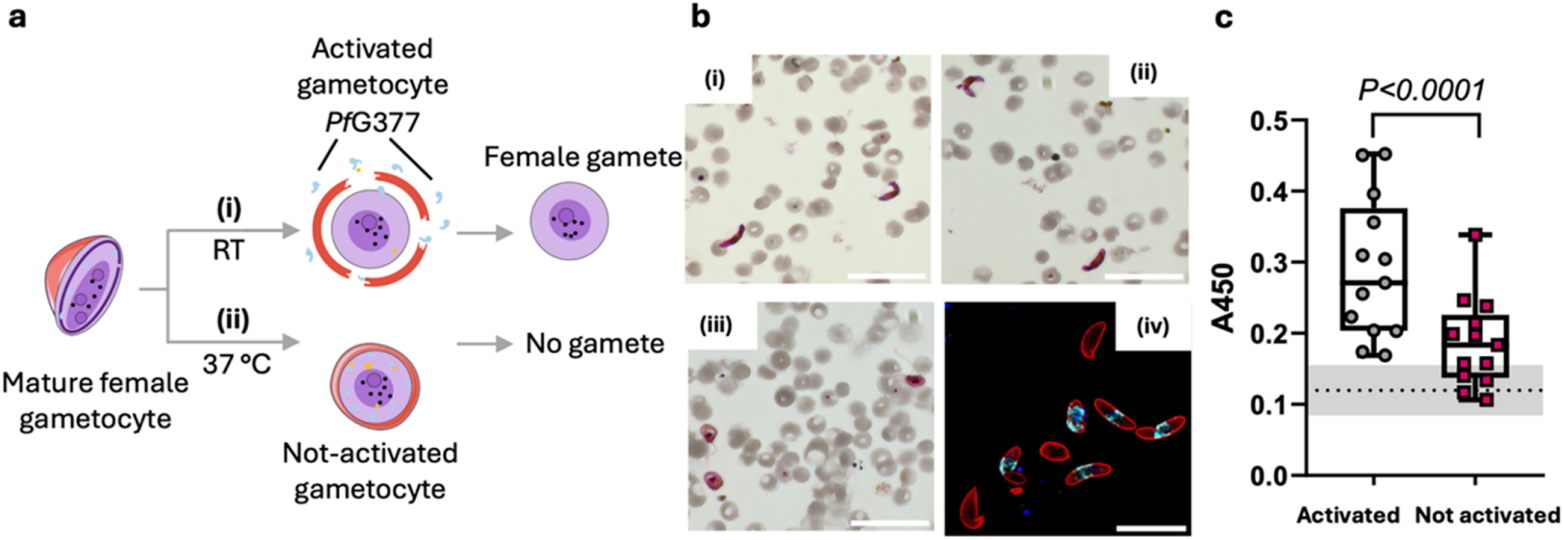
Detection of activatable female gametocytes in gametocyte culture samples through the detection of *Pf*G377. (a) Gametocyte activation can be artificially induced by a reduction in temperature. (i) Gametocytes incubated at room temperature of ∼21 °C for 1 h will activate and (ii) gametocytes incubated at 37 °C will remain inactivated. Schematic modified from Servier Medical Art Web site CC-BY 4.0. (b) Microscopy image of Giemsa-stained thin blood smears of mature stage V gametocyte cultures before (i/ii, crescent shapes) and after activation (iii, rounded forms), highlighting gamete release upon activation. Scale bars 30 μm. (iv) Z-projection of deconvolved widefield fluorescence image showing *Pf*G377 immunostaining using the same antibody used in the NLISA (red, Pfs16; cyan, *Pf*G377; blue, DAPI nucleus). Scale bar 15 μm. (c) Determination of gametocyte activation process through measurement of *Pf*G377 release (*N* = 13 independent cultures, *n* = 3 technical replicates each). The dashed line corresponds to the signal from the background with gray shades representing the s.d. of the background signal. Box and whisker plots represent the median and quartiles. Two-tailed paired Student’s *t* test statistical analysis was performed.

### *Pf*G377-NLISA Detects the Inhibitory Effect of Transmission-Blocking Drugs on Laboratory Mosquito Infection

In addition to detecting transmission-competent individuals within an endemic population, another critical role of malaria-elimination-focused diagnostic assays would be to monitor the effect of transmission-blocking drugs. The current gold standard is the detection of infected mosquitoes by skin feeding or a direct membrane feeding assay (DMFA). DMFA uses patient blood and laboratory-reared mosquitoes, which is extremely technical, time-consuming (7–10 days until mosquito dissection and counting), and difficult to standardize.^48^ Hence, a simple and rapid (few hours) diagnostic, such as an immunoassay-based test, would be very helpful for the evaluation of interventions on malaria transmissibility in patients under treatment during field trials or elimination settings. Toward this end, we next studied whether the addition of gametocytocidal drugs, which reduce mosquito infection by killing or inactivating gametocytes, correlates with lower *Pf*G377 measurements in our NLISA. MB and gentian violet (GV, also known as crystal violet) are dye molecules with high gametocytocidal activities that have been shown to fully inhibit the functional viability of both male and female gametocytes at concentrations of 10 and 20 μM, respectively.^7,49^ Since PQ is only active against mature *P. falciparum* gametocytes once metabolized in the liver,^50^ we could not use this drug in our *in vitro* assays, while MB is active as is and has also previously been shown to reduce transmission in clinical settings.^31^

We first confirmed the transmission-blocking ability of MB and GV by feeding drug-treated parasite cultures to laboratory mosquitoes in standard membrane feeding assays (SMFAs, Figure 4a). Ten days post feeding, mosquitos were dissected and the number of oocysts developed in their midgut was counted and reported as the mean oocyst intensity. To study the effect of gametocytocidal chemicals on the release of *Pf*G377 protein (Figure 4b), culture media containing MB at a concentration of 10 μM or GV at a concentration of 20 μM were prepared. *P. falciparum* stage V mature gametocyte cultures were then washed using either treatment media to remove any residual *Pf*G377 released due to accidental activation during gametocyte culturing. Washed gametocytes were then incubated in the GV or MB-containing treatment media at 37 °C for 24 h. This mimics the situation where a gametocyte-carrying patient would have taken a gametocytocidal drug that reaches their bloodstream. *Pf*G377 release was then detected, post *in vitro* activation, using our NLISA setup.

**Figure 4:**
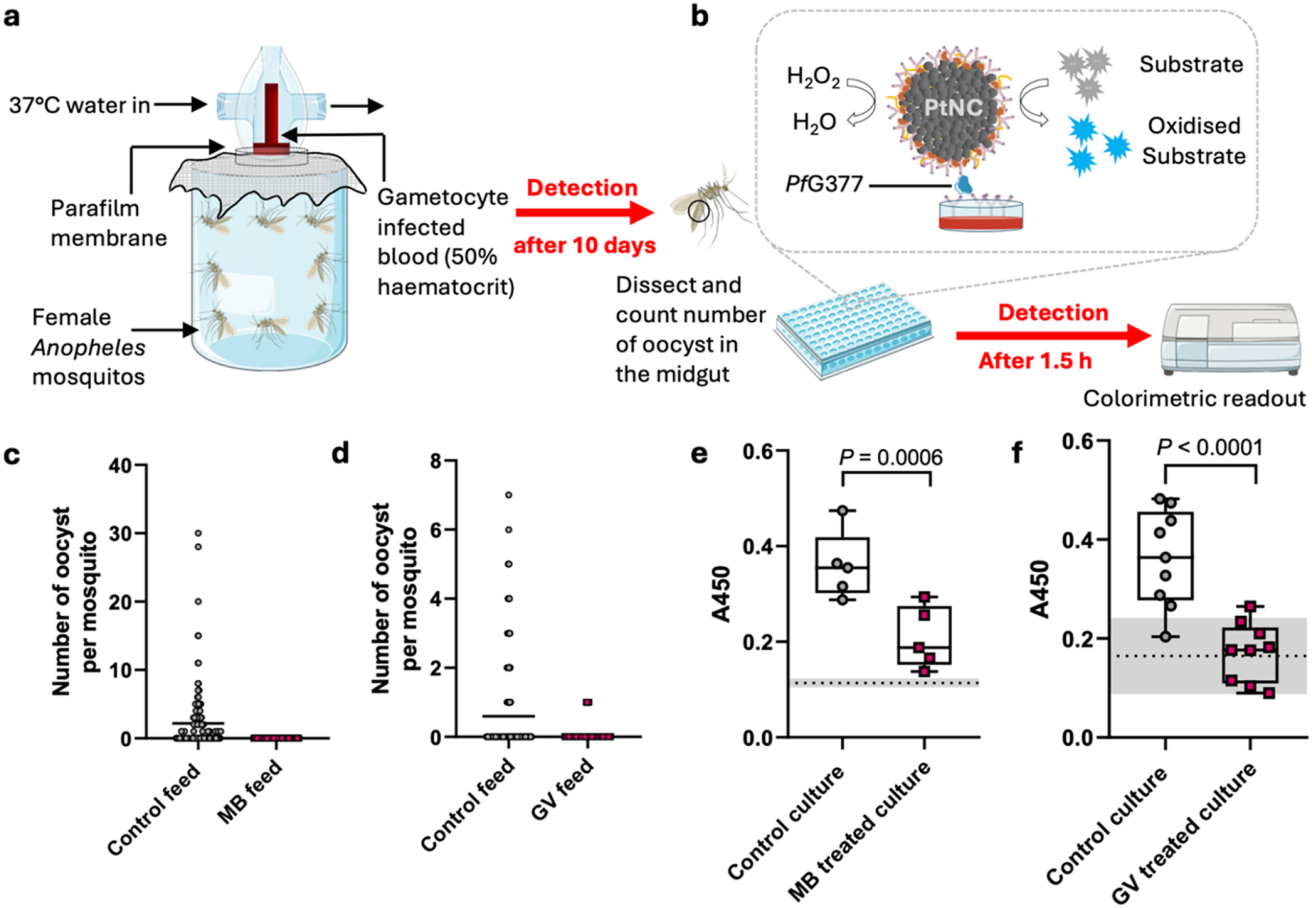
Assessment of *Pf*G377 immunoassay for evaluating the transmission competency for cultures before and after drug treatment compared to the standard membrane feeding assay (SMFA). (a) SMFA setup, the current gold standard method for determining malaria transmission reducing activity with laboratory cultures, testing the transmission-blocking effect of two gametocytocidal compounds, methylene blue (MB) and gentian violet (GV). (b) NLISA setup to detect released *Pf*G377 from gametocytes. Schematics (a, b) were modified from Servier Medical Art Web site CC-BY 4.0. (c, d) SMFA results were obtained 10 days after feeding untreated (control) and drug-treated gametocyte cultures to mosquitoes. A total of 52–71 mosquitoes were dissected per condition. Specifically, we performed 7 feeds for the MB control group, 10 feeds for the GV control group, and 3 feeds each for the drug-treated MB and GV cultures (all independent cultures). Horizontal bars indicate the mean. (e, f) Gametocyte cultures (± drug treatment; some control data points are used in both (e) and (f), as both drug treatments were performed simultaneously in some cases) were activated and assayed for *Pf*G377 by NLISA (*N* = 5 (MB) or 9 (GV) independent cultures, *n* = 3 technical repeats each). The dashed line corresponds to the signal from the background (respective drug + gametocyte media) with gray shades representing the s.d. of the background signal. Box and whisker plots represent median and quartiles. Two-tailed paired Student’s *t* test statistical analysis was performed.

Pretreating the gametocyte cultures with MB or GV solutions resulted in nearly complete prevention of malaria transmission to mosquitos when using SMFAs (Figure 4c,d), confirming previous data.^7,49,51^ The control feed, which contained healthy/untreated gametocytes, resulted in various degrees of infection in mosquitos, as indicated by the various densities of oocysts developed in mosquitos’ midguts. The results obtained from the *Pf*G377-NLISA platform agreed with the SMFA results and showed that the inhibition of gametocytes by GV and MB led to a reduction in the level of *Pf*G377 release in the activated samples. The drug-treated samples displayed a significantly lower absorbance signal in the NLISA assay compared to that of untreated gametocytes (Figure 4e,f). This difference suggests that the gametocytocidal activity of these drugs suppresses gametocyte activation and downstream *Pf*G377 release, confirming the suitability of *Pf*G377-NLISA in monitoring the effect of gametocytocidal interventions. Further testing of an expansive library of gametocytocidal compounds,^34,35^ with known and varied modes of action, as well as drug combinations would be important to assess the *Pf*G377-NLISA platform across a broad range of drugs with varied transmission-blocking modes of action. Accurately predicting the transmission potential by the NLISA platform without having to perform SMFAs could drastically simplify and speed up the monitoring of transmission-competent individuals, although finding a setup with appropriate specificity and sensitivity is the key challenge.

### Clinical Validation of the NLISA Platform Using Patient Samples from Endemic Regions

Application of a transmission sensor in a field setting would require taking a blood sample from the suspected parasite carrier (symptomatic or asymptomatic) and performing the NLISA developed herein after activation for 20 min to 1 h *ex vivo*. If mature gametocytes are present, they would activate upon a temperature drop accompanied by *Pf*G377 release, which can then be detected by NLISA (Figure 5). As a first step toward this clinical use case, we sought to determine whether malaria HRP2 RDT-positive versus RDT-negative patient blood samples can be distinguished with our NLISA platform. This first evaluation involved blood samples that were taken before treatment; hence, activation of gametocytes, if present, would be expected, but it did not include any study on transmissibility. We analyzed samples from 24 individuals living in the malaria endemic regions of Suhum, Kibi, and Koforidua in Ghana. Samples were classified beforehand as malaria positive or negative using commercially available HRP2-RDT (Abbott brand; gametocyte density ranged from 0 to 2000 gametocytes/μL, as estimated by microscopy).

**Figure 5:**
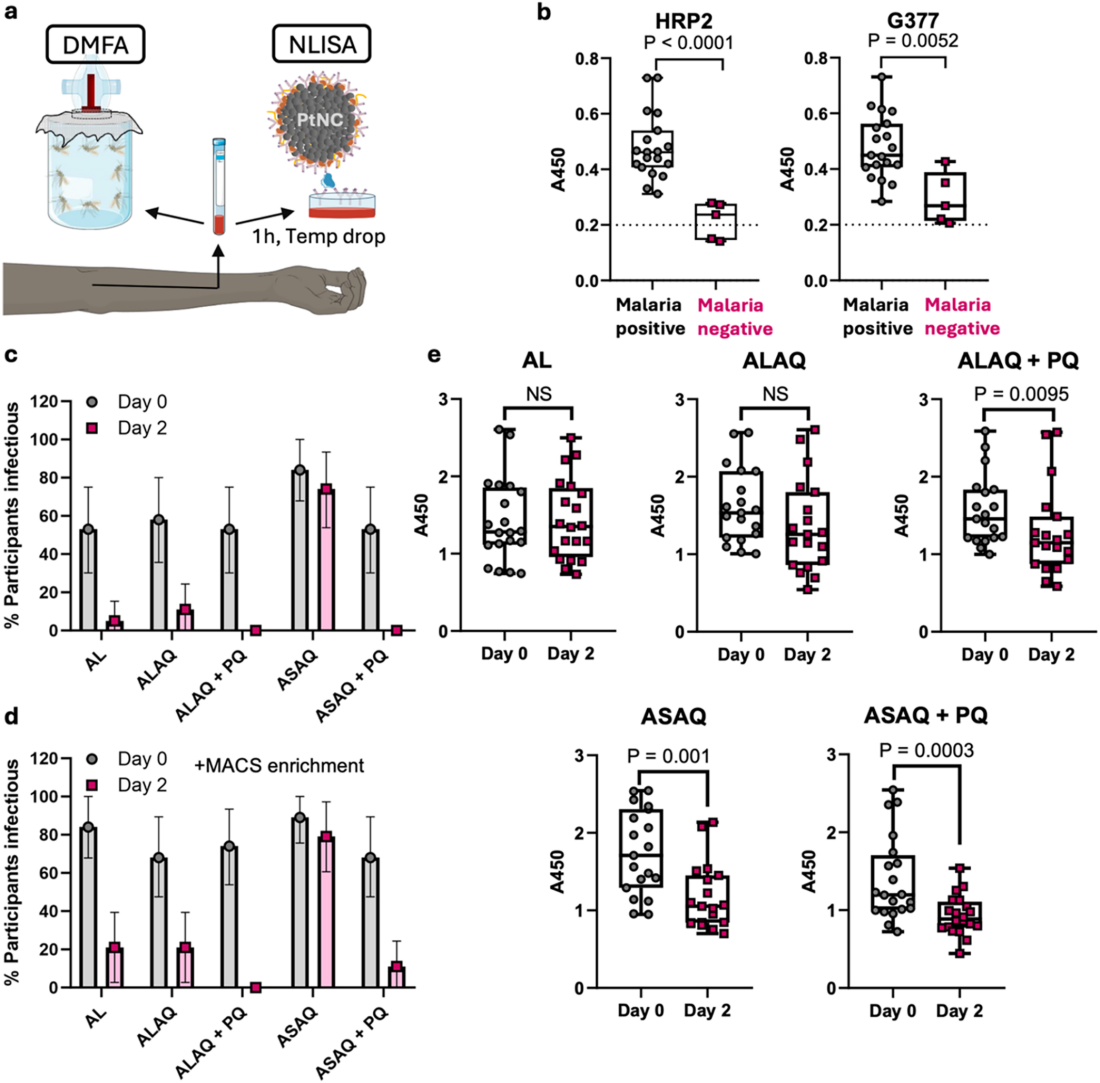
Clinical validation of the *Pf*G377-NLISA platform using patient samples from endemic regions. (a) Experimental setup for (c–e) comparing the *Pf*G377-NLISA signal from activated blood samples with the transmission potential of the same individuals using the direct membrane feeding assay (DMFA). Schematic modified from Servier Medical Art Web site CC-BY 4.0. (b) Initial validation of the HRP2 and *Pf*G377 NLISA platform with plasma samples from malaria positive and negative individuals (verified by commercial HRP2 RDTs) in Suhum, Kibi, and Koforidua, Ghana, without analyzing transmission competency (*N* = 24 patient samples, *n* = 3 technical replicates). Two-tailed Mann–Whitney statistical tests were performed. The dashed line indicates the background signal from uninfected control plasma samples. (c, d) Data from a previously published transmission trial in Mali, which shows % of infectious individuals that caused mosquito infection in DMFAs where (c) whole blood was tested for infectivity directly and (d) whole blood was tested directly and with gametocyte enrichment (magnet purification), both comparing pre- (day 0) and post-treatment (day 2) (*n* = 19–20 individuals per group). Error bars show 95% CI. AL = artemether-lumefantrine; ALAQ = artemether-lumefantrine-amodiaquine; ALAQ + PQ = artemether-lumefantrine-amodiaquine plus primaquine; ASAQ = artesunate-amodiaquine; ASAQ + PQ = artesunate-amodiaquine plus primaquine. Reprinted (Adapted) with permission from ref 33. Copyright 2025, Elsevier, CC-BY. (e) *Pf*G377 release assessment using the *Pf*G377 PtNC-NLISA platform in clinical samples from malaria-infected patients, comparing pre- and post-treatment with one of the five antimalarial treatments (*n* = 19–20 individuals per group). Box and whisker plots represent median and quartiles. Two-tailed Wilcoxon paired test statistical analysis was performed. NS refers to not significant.

When evaluating the presence of HRP2 and *Pf*G377 in these samples using our NLISA platforms, we found significant differences between the RDT-positive and -negative groups for both biomarkers (Figure 5b). This confirms that the result of our HRP2 NLISA agreed with the data obtained with the commercial asexual RDTs (initially used to classify the samples). The *Pf*G377 NLISA was also able to distinguish between the two groups, but no correlation between the observed NLISA signal and the density of gametocytes, as measured by microscopy, was seen. Moreover, we do not know from these experiments whether the biomarker was released in the human host (from normal gametocyte senescence or immune-mediated rupture) or immediately upon blood sampling. The *Pf*G377 signals above baseline in the malaria-negative group could have resulted from various reasons, including any *Pf*G377 that might have circulated in the bloodstream from a previous infection or an issue with assay specificity due to the polyclonal nature of the employed antibody in our current NLISA. The clinical sensitivity and specificity to diagnose malaria is higher for the HRP2 assay (100%, 100%, Figure S12) than for the *Pf*G377 assay in its current format (89.5%, 80%, Figure S13), which can potentially be linked to the use of high-affinity monoclonal antibodies (HRP2) versus polyclonal antibodies (*Pf*G377), highlighting the future possibility to improve the *Pf*G377 assay when using more specific high-affinity reagents. Nevertheless, this first clinical evaluation experiment encouraged us to next test whether *Pf*G377 detected in our NLISA can be related to the transmission potential.

A recent transmission trial evaluated the effect of various combination therapies with and without low-dose gametocytocidal PQ on onward transmission determined via DMFAs.^33^ A small aliquot of the blood samples was separated before the mosquito feeding and cooled down for 1 h to ensure gametocyte activation, if present, and subsequently evaluated with our *Pf*G377 NLISA. The study included five experimental groups (*n* = 19–20 individuals per group): artemether-lumefantrine (AL), artemether-lumefantrine-amodiaquine (ALAQ), artesunate-amodiaquine (ASAQ), and the latter two combined with low-dose PQ (ALAQ + PQ and ASAQ + PQ). The transmission trial data (published elsewhere)^33^ in terms of infectious individuals established via DMFAs at day 0 before drug treatment and at day 2 after treatment is reproduced as bar plots in Figure 5c. Overall, all the drug combinations lowered the number of infectious individuals to various degrees: ASAQ (little effect) < AL = ALAQ < ALAQ + PQ = ASAQ + PQ (0 transmission). Both PQ groups showed zero infectious individuals post-treatment (day 2), confirming the high transmission-blocking potential of a single low dose of PQ in combination with other antimalarials. DMFA typically gives lower mosquito infection than direct skin feeding assays (real-world transmission potential).^48^ Attempts to improve mosquito infection in DMFA include preconcentration of gametocytes by magnet purification after blood sampling before feeding. This was performed in the clinical study^33^ related to this paper, which confirmed that 2 days after drug treatment, ASAQ treatment had little effect on transmission, AL and ALAQ treatment only achieved partial inhibition of transmission, two individuals from the ASAQ + PQ group also acted as transmitters, while ALAQ + PQ completely blocked transmission (Figure 5d).

When studying the *Pf*G377-NLISA data for these patient samples after activation, we noted some key differences that were noteworthy. First, both + PQ groups showed significantly lower *Pf*G377 when comparing pre- (day 0) and post- (day 2) treatment blood samples. This agrees with our *in vitro* data that showed a lower release of *Pf*G377 upon treatment of gametocyte cultures with transmission-blocking gametocytocidal drugs (Figure 4). AL and ALAQ were not as potent in reducing transmission (Figure 5c,d) and did not show significant differences by NLISA when comparing days 0 to 2 for each treatment arm. This may be explained by high enough numbers of viable and activatable gametocytes remaining in these blood samples after drug treatment. The only outlier from this set of data is the ASAQ group, which showed a poor reduction in transmissibility but still significantly lower *Pf*G377 levels. There are several possible reasons that could possibly explain this result. First, this group had the highest gametocyte numbers and the smallest change of all groups, pre- and post-treatment (measured by PCR, Figure S14), and we also detected the highest mean *Pf*G377 signal for this group at day 0. At higher gametocyte numbers, a large fraction of gametocytes might have been inactivated by the drug, causing lower *Pf*G377 levels detected at day 2. However, there might have been a high enough fraction of viable gametocytes left to cause mosquito infection. Second, the ASAQ mode of action might have reduced *Pf*G377 expression in all the gametocytes, but they remained transmission competent. Hence, future studies that correlate the drug mode of action to *Pf*G377 in relation to transmissibility and efforts to define a threshold level for *Pf*G377 are warranted. This could include *in vitro* studies to evaluate *Pf*G377 expression levels at drug concentrations that do not completely kill the gametocytes to test the second hypothesis of lowered *Pf*G377 expression in drug-treated gametocytes at nonlethal levels. Addition of XA to our clinical samples, which is a major activator of gametocytes found in mosquitoes,^44^ is one way of improving our assay with patient samples. Using better affinity reagents, such as monoclonal antibodies, nanobodies, or affibodies, specific for *Pf*G377 could also help to improve the sensitivity and specificity of our NLISA. However, the polymorphic nature of *Pf*G377, especially region 3,^52^ will have to be considered when selecting fragments for the production of high-affinity reagents. Another option would be to develop similar assays for alternative antigens that were previously identified in the supernatant after gametocyte activation.^13,14^ In summary, our *Pf*G377-NLISA displayed a lower signal intensity for the groups with the lowest transmission potential (+ PQ), highlighting the potential of such an assay to be developed into an RDT that can be used in various transmission settings.

## Conclusions

Limiting parasite transmission is key to malaria control and eventual eradication. Transmission reduction through the application of transmission-blocking drugs may be made more effective by targeting individuals and groups with high transmission potential, many of which may be asymptomatic carriers. Development of a blood-based RDT to detect viable *Plasmodium* gametocytes, similar to widely used HRP2-based RDTs for infection detection, could make the detection of individuals with transmission competent infections more scalable and simplify the implementation and community acceptance of surveillance for transmission-focused interventions. Currently employed RDTs detect antigens, such as HRP2, of asexual *P. falciparum*. A major limitation of these RDTs, especially in the context of malaria transmission and elimination, is their relatively low sensitivity and sole focus on asexual parasites.^53,54^ Gametocytes also produce low levels of HRP2, but the amount and their relative biomass make their contribution to standard RDT-relevant antigenemia (specifically HRP2) negligible.^29^ Even with recently developed ultrasensitive RDTs detecting HRP2 at very low levels, it is not yet possible to detect the whole reservoir of parasite carriers, including asymptomatic individuals.^24,55^ Further, HRP2 can be detected up to 33 days after clearing an infection, which can result in false positive detection.^29^ More worryingly, several studies in East Africa have now reported widespread loss of HRP2 from parasites, such strains can easily escape detection by current HRP2-based RDTs.^56,57^ These results highlight that enhancing the LoD is not the only challenge with the current RDTs.

Here, we developed a nanozyme-based immunoassay for the detection of female gametocyte-specific *Pf*G377, which is released after gametocyte activation upon blood feeding by mosquitoes or blood sampling. Female gametocytes that fail to release *Pf*G377 upon activation would be predicted to be incapable of gametogenesis and by extension onward transmission, given the importance of OBs in successful gametogenesis and transmission.^9^ Hence, the absence of this biomarker in a blood sample after activation was hypothesized to be linked to lower transmission competence. To verify this hypothesis, we first demonstrated optimization of the NLISA using recombinant antigen as the target. We then showed that cultured *P. falciparum* gametocytes release significant amounts of *Pf*G377, which was detected with our NLISA after gametocyte activation. Pretreatment of the cultures with known gametocytocidal compounds (MB and GV) reduced the amount of released *Pf*G377, indicating *Pf*G377 retention inside drug-inactivated gametocytes, since this protein was already expressed since stage III, well before the drug treatment. In clinical patient samples, the *Pf*G377-NLISA successfully distinguished infected from noninfected individuals. Further, upon treatment of patients with antimalarial combination therapies containing low-dose PQ, which nearly completely abolished transmission to mosquitoes in DMFAs, lower levels of *Pf*G377 were detected. Nevertheless, another non-PQ drug combination showed reduced *Pf*G377 even though mosquito transmission was still prevalent. This indicated that starting gametocytemia and the drug mode of action are important considerations to be taken into account in future studies. Future work is required to improve assay sensitivity and specificity through use of high-affinity recognition elements, such as monoclonal antibodies, and improve nanozyme catalytic activity to be implemented in the NLISA design. The assay can then be transferred to a paper-based LFIA^36–39^ to serve as a RDT for transmission competence, which we have started herein by successfully performing half-dipstick assays with our *Pf*G377-PtNCs. Any plasma component, such as hemoglobin from hemolysis, that could potentially catalyze TMB oxidation, would be removed in the final lateral flow-based setup (not retained at the test line). The signal would be generated by PtNCs that are retained at the test line only in the presence of the target antigen. Future clinical sample analysis should also incorporate blood sample purification under nonactivating conditions to establish whether any *Pf*G377 is already present in the bloodstream of malaria parasite carriers in the absence of activation. If *Pf*G377 is present, it needs to be established how long it circulates after clearing an infection, as has been done for HRP2 previously.^25^ Next, finding the correct thresholds for the assay or implementing a simple purification step could solve the issue of potential background *Pf*G377 in the blood. Overall, the development of a sensitive and specific RDT to detect transmission-competent individuals through the detection of antigens released from activated gametocytes could be an important tool for drug development and triage in field settings and could be added to current and future malaria elimination trials and strategies.

## Materials and Methods

### Reagents and Instruments

Most reagents, if not specified differently, were obtained from Sigma-Aldrich (U.K.). For NLISA signal generation we either used a homemade TMB substrate solution consisting of a 250:4:1 ratio of 50 mM citrate buffer (pH 5.0), TMB (6 mg/mL in dry DMSO), and 5 wt % H_2_O_2_ or we used commercial TMB X-tnd ELISA HRP substrate solution from Kementec with no adjustments. Anti-HRP2 Monoclonal Antibody 6C8 clone (catalogue no. CAT-002-06-005-02) was purchased from Precision antibody. Anti-*Plasmodium falciparum* HRP2 Antibody (Mouse IgM)—Monoclonal clone MPFM-55A was bought from 2BScientific. Anti-*Pf*G337 polyclonal antibodies (Rabbit IgG) and recombinant *Pf*G377 were produced as described elsewhere.^11^ Protein high-binding and dilution plates were purchased from Corning (Corning Ltd., UK). HiTrap Protein G HP antibody purification columns (1 mL, catalog no. 17-0404-03) were purchased from Cytiva. Amicon Ultra 0.5 mL centrifugal filter units (Merck) were used for buffer exchange and antibody concentration. Washing steps were performed on a Biotek ELx465 automated plate washer (Biotek Inc.). Absorbance was read on a SpectramaxPlus instrument (Molecular Devices, Sunnyvale, CA, USA). Protein concentration was measured on a NanoDrop 2000c spectrophotometer (Thermo Scientific).

### Gold Nanoparticle Seed Synthesis

Spherical gold nanoparticle seeds of size ca. 15 nm in diameter were prepared by reduction of HAuCl_4_ with sodium citrate.^42^ In a typical synthesis, a 5 mL gold­(III) chloride trihydrate aqueous solution (40 mM, Sigma) was added to 175 mL of ultrapure deionized water (Invitrogen) under reflux at 100 °C with vigorous stirring. Nanoparticle synthesis was then initiated by fast injection of 10 mL of trisodium citrate dihydrate solution (2 wt % in water, Sigma) with vigorous stirring and refluxed for 15 min. Reaction completion was confirmed when the solution turned from an initial light gold to a dark red color. The resulting gold nanoparticles (AuNPs) were cooled to room temperature and stored at 4 °C. AuNPs were characterized by TEM size analysis, and the concentration was determined through UV–vis spectrometry.

### Porous Platinum Core–Shell Nanoparticle (PtNC) Synthesis

Urchin-like PtNCs of ca. size 120 nm in diameter were synthesized by reduction of chloroplatinic acid hydrate on gold seeds adapted from Loynachan et al.^36^ In a typical synthesis, 170 μL of AuNP seeds from above (18 nM) was added to 9.8 mL of ultrapure deionized water (Invitrogen). After stirring the solution at 750 rpm for 2 min, 200 μL of PVP (10 kDa, 20 wt % in ultrapure deionized water, Sigma) was added, and the solution was gently stirred at 60 rpm for 30 min. Then, 400 μL of l-ascorbic acid (100 mg/mL, Sigma) and 400 μL of chloroplatinic acid hydrate (100 mM, Sigma) were added sequentially and mixed briefly. The solution was then stirred (400 rpm) and heated to 80 °C in an oil bath for 45 min. A color change from red to black indicated the successful deposition of platinum. The PtNC suspension was then cooled to room temperature for purification by four sequential centrifugation (1250 rcf for 12 min) and resuspension steps using ultrapure deionized water.

### Nanoparticle Characterization

A SpectraMax M5 multimode microplate reader (Molecular Devices Ltd.) was used for the absorbance measurements. A Zeta Sizer (Nanoseries, Malvern Instruments Ltd.) was used to characterize the nanoparticle hydrodynamic diameter and zeta potential. Samples for electron microscopy were drop-cast onto carbon-coated copper grids (Electron Microscopy Sciences) and left to dry before imaging by TEM on a JEOL 2100F operating at 200 kV. Scanning transmission electron microscopy (STEM) mode was used to acquire high-angle annular dark-field (HAADF) images using a camera length of 10–12 cm (HAADF5) on the JEOL 2100F. Energy-dispersive X-ray spectroscopy (EDS) in STEM mode was used for elemental compositional mapping on PtNCs.

### Antibody Purification from Antiserum

Antibody purification from antiserum was performed in two steps: (1) salting out using a saturated ammonium sulfate solution and (2) purification using HiTrap Protein G HP antibody purification column (Cytiva, catalogue number 17-0404-03). Ammonium sulfate precipitation was carried out as follows: *Pf*G377 rabbit antiserum (600 μL) was cooled to 0 °C in an ice bath while gently stirring. Ammonium sulfate (419 μL, 4.1 M at 25 °C equivalent of 100% saturated solution) was added dropwise to this solution to achieve a final concentration of 41% saturation of (NH_4_)­SO_2_. The solution was stirred gently for 4 h at 0 °C. The precipitated solution was centrifuged for 15 min at 17500 rcf, the supernatant was discarded, and the white precipitate was redissolved in 200 μL of binding buffer (20 mM sodium phosphate, pH 7.0). Protein G purification was done as follows: 1 mL column was first washed with 10 mL of binding buffer (20 mM sodium phosphate, pH 7.0) to set the pH of the column to 7.0, ready for antibody binding. The solution obtained from ammonium sulfate precipitation was then filtered through a 0.45 μm Millex-Gv nonpyrogenic low protein binding filter (Sigma) and slowly loaded onto the column. 7 mL of binding buffer was added to the column to wash off any unbound proteins. And the column was then eluted with the addition of 10 mL elution buffer (0.1 M glycine-HCl, pH 2.7). Eluted fractions were then collected, and their protein concentration was measured using NanoDrop. The fractions containing >0.5 mg/mL antibody were all pooled together, buffer-exchanged into PBS, and concentrated using an Amicon 10 kDa filter.

### PtNC-Antibody Conjugate Preparation

A typical conjugation reaction contained 3.6 μL of antibody solution (6.7 μM) mixed with 20 μL of HEPES buffer (pH 7.0, 0.1 M), followed by the addition of 200 μL of the prepared PtNCs (120 nm, 300 pM) in ultrapure deionized water. The solution was briefly vortexed and incubated for 3.5 h under shaking (500 rpm) at room temperature for antibody attachment through physisorption. Then, 200 μL of β-casein from bovine milk (2 wt % in PBS, Sigma) was added to block the remaining free spaces on the nanoparticle’s surface. The solution was incubated for a further 1.5 h under shaking at 800 rpm at room temperature. Modified particles were then purified through 3 washing cycles at 1250 rcf for 12 min in assay running buffer (0.2 wt % β-casein, 0.2 wt % Tween-20 (Sigma) in PBS pH 7.0). Particles were eventually resuspended in the assay running buffer and stored at 4 °C until use.

### The Checkerboard Titration NLISA

To optimize the design of the sandwich NLISA, a 2D indirect NLISA was used as follows. A serial dilution of capture antibody (5 to 0.078 μg/mL and zero in carbonate buffer pH 9.6, 100 μL/well) was first coated onto a protein high-binding 96-well microtiter plate overnight at 4 °C and covered with an adhesive plate sealer. The next day, the plate was washed three times with phosphate-buffered saline with 0.05 wt % Tween 20 (PBST, 300 μL per well). Antigen at a concentration of 100 ng/mL was added to all wells (100 μL per well). After a 30 min incubation with the antigens, the plate was washed with PBST (300 μL per well). Next, a serial dilution of detection antibody-PtNC complex (1.5 to 0.38 pM and zero in PBST buffer pH 7.0 for HRP2 or 3 to 0.19 pM and zero in PBST buffer pH 7.0 for *Pf*G377) was added to the plate. After a 30 min incubation period at r.t., the plate was washed 3×, and the TMB substrate solution was added (100 μL per well). Color development was stopped after 30 min at r.t. with 4 N H_2_SO_4_ (50 μL per well), and absorbance was read at 450 nm using a plate reader.

### Sandwich NLISA

A 384-well microtiter plate (384 wells, clear polystyrene Maxisorb, Nunc) was first coated with the capture antibody (0.75 μg/mL for HRP2, and 2 μg/mL for *Pf*G377 in carbonate buffer pH 9.6, 100 μL per well) overnight at 4 °C, covered with an adhesive plate sealer. The next day, the plate was washed three times with PBST (100 μL per well), and the solution of serial dilution of antigen (starting from 300 ng/mL and diluting by a factor of 1/4 in PBST, 100 μL per well) was added to the plate. After a 30 min incubation, the plate was washed three times with PBST (100 μL per well). Antibody-functionalized PtNC detection unit was then added (1 pM for HRP2, and 2 pM for G377 system in PBST buffer pH 9.6, 100 μL per well). After a 30 min incubation period at r.t., the plate was washed 3×, and the TMB substrate solution was added (100 μL per well). Color development was stopped after 30 min at r.t. with 4 N H_2_SO_4_ (50 μL per well), and absorbance was read at 450 nm using a plate reader.

### Half-Dipstick Immunochromatographic Assay Performance Evaluation

To prepare the immunochromatographic assay (ICA) strips, antibodies were printed on the nitrocellulose membrane (CN95 Unisart Nitrocellulose Membrane, Sartorius) using the BioJet automated liquid dispenser system (BioDot Inc.) at a dispense rate of 1 μL/cm. 0.5 mg/mL of filtered (0.2 μm filter) anti-*Pf*G377 polyclonal antibody and goat anti-rabbit IgG H&L (ab6702) was dispensed at a height of 5 mm (test line) and 13 mm (control line) from the bottom of the nitrocellulose paper, respectively. The printed membranes were then fully dried overnight in a 37 °C oven. Finally, the dried antibody-printed nitrocellulose membranes were assembled onto the backing card (Kenosha, KN-PS1060.19) with overlapping absorbent pad material (Ahlstrom-munksjo, KN-222-20.1), before being cut into 4 mm-wide test strips.

PtNC-antibody conjugate detection probes used here were prepared by conjugating the PtNCs with *Pf*G377 antibodies as reported above, using a ratio of 400 Ab/PtNC. The PtNC-antibody conjugates were then resuspended in running buffer (0.2 wt % β-casein, 0.2 wt % Tween-20 (Sigma) in PBS pH 7.0) to a final concentration of 150 pM. ICA strips were run in half-dipstick format by dipping the test strips into a well of a 96-well plate (Corning no. 3641, flat bottom, nonbinding surface) containing the PtNC-antibody conjugate (15 μL at 150 pM in running buffer) detection probe along with recombinant *Pf*G377 spiked into fetal bovine serum (FBS, Gibco, Thermo Fisher) to reach a final volume of 65 μL in the well. After the solution had fully wicked up the strip (around 10 min), the strip was then dipped in another well containing 100 μL of running buffer (0.2 wt % β-casein, 0.2 wt % Tween-20 (Sigma) in PBS pH 7.0) for 10 min to wash through any unbound nanoparticles or loosely bound nonspecific proteins. Subsequently, the strip was submerged for 5 min in a 1.5 mL amber Eppendorf tube filled with 1000 μL freshly prepared PtNC substrate solution containing a modified CN/DAB (4-chloro-1-naphthol/3,3′-diaminobenzidine, tetrahydrochloride) substrate kit (Thermo Scientific) adjusted with hydrogen peroxide solution 30% (w/w) (Sigma) to reach a final added peroxide concentration of 4 M. Finally, the strip was moved into a 1.5 mL protein loBind tube (Eppendorf) containing 1000 μL ultrapure distilled water (Invitrogen) for 3 min to stop the reaction. The strips were then briefly dried under ambient conditions for ease of handling.

### Strip Analysis

Strips were imaged 20 min after removal from water using an iPhone 11 ProMax mobile phone camera. Test line intensities were quantified using ImageJ software by first converting the image to grayscale (16 bit) before drawing a rectangle corresponding to the width of the lateral flow strips and length long enough to include an internal control of the strip (control line). Using the gel analyzer tool, the pixel density of each test line was integrated. To determine the detection limit of the assay, the pixel intensity was plotted against the antigen concentration in a log–linear calibration curve, which was fitted to a four-parameter equation using GraphPad Prism 8.1.0 (GraphPad Software Inc., San Diego, CA, USA), according to the formula in the analysis section.

The background pixel intensity is measured using ImageJ software by assessing the background pixel intensity of all of the strips in a single serial dilution calibration assay and calculating the average of these values.

### Gametocyte Culture Induction and Maintenance

*P. falciparum* gametocytes were induced and cultured as previously reported,^7^ using the NF54 and NF135 strains from Africa and Southeast Asia, respectively. Briefly, gametocytes were induced from asexual blood-stage cultures at 2.5% parasitemia and 5% hematocrit. Gametocytes were grown in gametocyte media, which is RPMI-1640 media with 25 mM HEPES (Life Technologies) supplemented with 2 g/L sodium bicarbonate (Sigma), 50 μg/L hypoxanthine (Sigma), 0.3 g/L l-glutamine, 0.5% (v/v) AlbuMAX II (Life Technologies), and 5% (v/v) A+ human serum (Interstate Blood-Bank). Following induction, daily gametocyte medium changes were performed for 14 days (no fresh erythrocytes added). 14 and 16 days post-induction, thin blood smears and Giemsa staining as well as determining the percentage of exflagellation centers relative to the total erythrocyte density were used to verify the presence of viable and activatable stage V gametocytes. Exflagellation was measured by activating the cultures by adding a 1:1 v/v ratio of culture medium to ookinete medium (same as the gametocyte culture medium explained above but without serum or AlbuMAX II and supplemented with 100 μM xanthurenic acid (XA)). Exflagellation centers were quantified by manual counting on a hemocytometer (VWR) under a Nikon Leica DC500 microscope.

### Immunofluorescence Staining and Imaging

The immunofluorescence staining and imaging was performed according to the paper by Yahiya et al.^58^ To prepare parasites for immunofluorescence staining and microscopy, *P. falciparum* NF54 mature stage V gametocytes were fixed with paraformaldehyde (prewarmed to 37 °C to prevent activation) to a final concentration of 4% (v/v) in PBS. Poly-l-lysine (Sigma)-coated glass coverslips were used to adhere the fixed samples, which were then washed once in PBS, permeabilized in 0.1% (v/v) Triton-X100, washed three times with PBS, and blocked with 10% (v/v) fetal bovine serum. Labeling with primary antibodies was performed for 1 h using the following dilutions: 1:800 mouse anti-Pfs16 clone 32F717:B02194 (a kind gift from Robert Sauerwein, Radboud University Medical Centre) and 1:800 rabbit anti-*Pf*G377 polyclonal antibodies.^11^ Labeling with secondary antibodies was performed for 45 min using the following dilutions: 1:500 anti-rabbit Alexa Fluor 488 (Thermo Fisher), anti-mouse Alexa Fluor 594 (Thermo Fisher), 10 nM 4′,6-diamidino-2-phenylindole (DAPI), and 5 μg/mL Wheat Germ Agglutinin (WGA)-633. Coverslips were mounted onto glass slides by using VectaShield (Vector Laboratories). A Nikon Ti-E inverted widefield microscope (NIS Elements v4.20 software) with a 100× objective was used to acquire images in 0.2 μm z-increments. EpiDemic plugin in Icy Bioimage Analysis software was used to deconvolve the z-stack images, and Fiji was used to create the composite figure as maximum intensity projections.

### Asexual Culture Preparation and Maintenance

*P. falciparum* strain 3D7 was cultured^59^ in human O+ RBCs at 2% hematocrit and 1% parasitemia using RPMI-HEPES (Sigma) medium supplemented with 0.292 g/L l-glutamine, 5 g/L AlbuMAX II (Gibco),^60^ 0.025 g/L gentamicin, and 0.05 g/L hypoxanthine.^61^ 5% (wt/vol) sorbitol was used for synchronization.^62^ Parasites were cultured at 37 °C in a gas mixture of 5% CO_2_, 5% O_2_, and 90% N_2_. To prepare a sample, parasites were prepared at 2% hematocrit and 1% parasitemia at the trophozoite stage in fresh medium and incubated overnight. The next morning, 5–6% mostly ring-stage parasites were obtained, the culture was spun down and the supernatant was subsequently harvested and sterile filtered, ready to be tested on the NLISA platform.

### *In Vitro* Activation of Gametocytes and Detection of *Pf*G377 by NLISA

Release of *Pf*G377 from activated stage V gametocytes was measured using the developed NLISA immunoassay. One mL of gametocyte culture containing gametocytes at day 16 post-induction was washed once in gametocyte medium at 37 °C to remove any *Pf*G377 that might have been released during the culture process. The pellet was then resuspended to half its original volume in gametocyte medium, and a serial dilution of the culture in gametocyte medium was prepared at 38 °C. Once the serial dilution was completed, it was transferred to room temperature, and the dilutions were all activated simultaneously by adding an activation medium (1:1 vol/vol ratio of culture: ookinete medium). Twenty minutes post-addition of activation medium, the amount of released *Pf*G377 from the activated gametocytes was measured on the NLISA platform.

The activated gametocyte samples were either added directly to the NLISA well plate (this form of sample is referred to as cell suspension) or were first centrifuged down, and the supernatant was collected and added to the NLISA well plate (this form of sample is referred to as supernatant). The degree of activation was further analyzed through counting the exflagellation centers of the gametocyte culture with a hemocytometer (VWR) using a Nikon Leica DC500 microscope.^7^

The NLISA was performed by coating a 384-well plate (384 wells, clear polystyrene Maxisorb, Nunc) with the capture antibody (at concentrations of 0.75 μg/mL for HRP2, 3 μg/mL for *Pf*G377 in carbonate buffer pH 9.6, 100 μL per well) overnight at 4 °C, covered with an adhesive plate sealer. The next day, the plate was washed three times with PBST (100 μL per well), and the solution containing the activated gametocytes was added to the plate (100 μL per well). Gametocyte culture medium was used as the negative control sample. After 30 min incubation, the plate was washed three times with PBST (100 μL per well). Antibody-functionalized PtNC detection unit was then added (1 pM for HRP2, and 2 pM for G377 system in PBST buffer pH 9.6, 100 μL per well). After a 30 min incubation period at r.t., the plate was washed 3x, and the TMB substrate solution was added (100 μL per well). Color development was stopped after 30 min at r.t. with 4 N H_2_SO_4_ (50 μL per well), and absorbance was read at 450 nm using a plate reader.

### Quantification of Exflagellation Density

The exflagellation density was quantified using the protocol developed by Delves et al.^7^ Briefly, the gametocyte culture was first washed and resuspended in warm gametocyte media. Next, 10 μL of the resuspended culture was transferred to a 1.5 mL tube prewarmed at 37 °C. The solution was then transferred to room temperature, and immediately 10 μL of ookinete medium (RPMI-HEPES supplemented with 100 μM xanthurenic acid, 200 μM hypoxanthine, and 10% (v/v) bovine serum albumin, pH 7.4) was added and thoroughly mixed to induce gamete formation. After 15 min, the solution was transferred to a Neubauer chamber, and exflagellation was observed by phase contrast microscopy with a 40× objective. The exflagellation centers per milliliter of culture were then calculated using the following formula:

$$\mathrm{Mean exflagellation of}\;4\;\mathrm{grids}\times 2\;\left(\mathrm{dilution factor}\right)\times 10^{4}=\mathrm{culture exflagellation per mL}$$

Next, the phase-bright RBCs were counted in 16 small squares of the central grid of the Neubauer chamber. Using the following formula, the number of RBCs per mL of culture was then calculated:

$$\mathrm{Mean RBCs of}\;16\;\mathrm{small squares}\times 100\times 2\;\left(\mathrm{dilution factor}\right)\times 10^{4}=\mathrm{RBC per mL}$$

Finally, the exflagellation density was calculated as the number of exflagellation cells as a percentage of the total cells according to the following formula:

(Culture exflagellation
per mL/RBCs per mL)×100=%exflagellation
cells

### Standard Membrane Mosquito Feeding Assay (SMFA)

Standard conditions were used to rear *Anopheles stephensi* mosquitoes (26:28 °C, 65–80% relative humidity, 12:12 h light/darkness photoperiod). Adults were typically maintained on 10% fructose. However, 12 h before an infectious blood meal, mosquitoes were starved. Previously described membrane feeding assays (SMFAs) were performed to infect mosquitoes.^63^ In brief, gametocyte cultures at days 15–17 containing at least 2% stage V gametocytemia were centrifuged down, the supernatant removed, and the pellet redispersed in a 50% (v/v) mixture of A + human serum and fresh RBCs. The target gametocytemia level was 0.1% for this final mix, which was subsequently used to feed starved female mosquitoes for 12 min. The feed mosquitoes were then kept without fructose for 24 h, which allows only mosquitoes that have been blood-fed to survive. Subsequently, mosquitoes were given fresh fructose daily up to the dissection day. Ten days postfeed, mosquitoes were dissected, and infection prevalence and intensity were then calculated by manually counting the oocysts per mosquito midgut.

The GV-treated medium was prepared by spiking 10 μL of GV stock solution (20 mM in DMSO (Sigma)) in 10 mL of gametocyte medium. The MB-treated medium was prepared by spiking 10 μL of MB stock solution (10 mM in DMSO (Sigma)) in 10 mL of gametocyte medium. Gametocytes were then treated with the drug of choice on day 15 post-induction by washing once with the prewarmed drug-treated medium or drug-free medium (control) and incubated at 37 °C for another 24 h. Then the cultures were spun down and washed once in the warm drug-treated or drug-free gametocyte medium. For NLISA measurement, the gametocyte cultures were transferred to room temperature and activated by activation medium (1:1 vol/vol ratio of culture: ookinete medium). Twenty minutes post-addition of activation medium, the amount of released *PfG377* from the activated gametocytes was measured on the NLISA platform. For SMFA, the gametocyte culture was diluted in a 50% (v/v) mixture of fresh RBCs and A+ human serum and then used to feed starved female mosquitoes as described above.

### Clinical Validation

Plasma samples from 24 individuals from Suhum, Kibi, and Koforidua in Ghana were collected for evaluation on the NLISA platform. Samples were all classified as malaria positive (presence of *P. falciparum*) or negative (lack of *Plasmodium falciparum*) using HRP2-based rapid diagnostic test (Abbott brand) as well as PCR. Giemsa-stained thin films were used to measure the number of gametocytes present in the samples. NLISA was performed with undiluted plasma samples. The sample collection and NLISA assays were all carried out at the West African Centre for Cell Biology of Infectious Pathogens (WACCBIP) of the University of Ghana. The study obtained ethical approval from the ethics committees of the Ghana Health Service (GHSERC005/12/17), the Noguchi Memorial Institute for Medical Research, the University of Ghana (NMIMR-IRB CPN 077/17-18), and the Kintampo Health Research Centre (KHRCIEC/2018-10). All participants and/or parents or guardians of participants gave written informed consent prior to recruitment.

Samples from the Malian transmission-blocking drug trial are derived from a phase 2 randomized controlled trial conducted at the Ouélessébougou Clinical Research Unit of the Malaria Research and Training Centre (MRTC) of the University of Sciences, Techniques and Technologies of Bamako in Mali (primary outcomes published elsewhere).^33^ In brief, 100 asymptomatic individuals aged between 10 and 50 years were enrolled, all of whom were gametocyte positive by microscopy (i.e., ≥1 gametocytes observed in a thick film against 500 white blood cells (WBC), equating to 16 gametocytes/μL with a standard conversion of 8000 WBC/μL blood). The 100 individuals were randomly allocated to five treatment groups in a 1:1:1:1:1 ratio (artemether-lumefantrine (AL), artemether-lumefantrine-amodiaquine (ALAQ), artemether-lumefantrine-amodiaquine plus primaquine (ALAQ + PQ), artesunate-amodiaquine (ASAQ), and artesunate-amodiaquine plus primaquine (ASAQ + PQ)). PQ treatment included a single dose of 0.25 mg/kg PQ (ACE Pharmaceuticals, Zeewolde, The Netherlands), which was administered at baseline in parallel with the first dose of schizonticidal treatment.^31^ Male and female gametocytes were quantified at any time point by using a multiplex reverse transcriptase quantitative PCR (RT-qPCR) assay (supplementary Tables 1–8 in ref 33).^15^ To assess infectivity, ∼75 locally insectary-reared female *Anopheles gambiae* mosquitoes were allowed to feed on venous blood samples (Lithium Heparin VACUETTE tube, Greiner Bio-One, Kremsmünster, Austria) for 15–20 min using a prewarmed glass membrane feeder system (Coelen Glastechniek, Weldaad, The Netherlands). Any mosquitoes that had only taken a partial blood meal or no blood meal were discarded. On the seventh day post-feeding, the surviving blood-fed mosquitoes were dissected. 1% mercurochrome was used to stain midguts. The presence and density of oocysts were then examined by expert microscopists. A separate blood sample (from baseline and day 2 only) was processed by magnetic-activated cell sorting (MACS), using a QuadroMACS separator and LS MACS columns (MiltenyiBiotech, U.K.) as previously described,^64^ to enrich its gametocyte content prior to mosquito feeding for transmission assays. Small aliquots of the venous blood samples on day 0 and day 2 were separated before mosquito feeding and allowed to cool for 1 h by keeping at room temperature (air-conditioned room, approximately 21 °C), the sample spun down in a centrifuge to remove the cells, and the supernatant was collected and frozen for subsequent analysis by NLISA. NLISA was performed with undiluted plasma samples. Ethical approval was granted by the Ethics Committee of the University of Sciences, Techniques, and Technologies of Bamako (Bamako, Mali) (No2022/244/CE/USTTB), and the Research Ethics Committee of the London School of Hygiene and Tropical Medicine (London, United Kingdom) (LSHTM Ethics ref 28014).

### Analysis and Statistics

Calibration curves were fitted to a four-parameter logistic equation using GraphPad Prism 8.1.0 (GraphPad Software Inc., San Diego, CA, USA). The 0 ng/mL is offset to allow transforming the data into a log format. The NLISA titration data was then plotted as a log–linear calibration curve, which was fitted to a four-parameter equation according to the following formula:

$$y=A_{\min}+\left(A_{\max}-A_{\min}\right)/\left(1+10^{\left(\left(\log{IC}_{50}-x\right)\times HillSlope\right)}\right)$$

where *A*_max_ is the maximal absorbance, *A*_min_ is the minimum absorbance, IC_50_ is the concentration producing 50% of the maximum absorbance, and Hillslope is the slope at the inflection point of the sigmoidal curve. The detection limit was selected as the antigen’s concentration giving rise to a signal equal to 10% of the maximum absorbance signal. The detection limit for the curves that do not reach saturation (Figure S11) was calculated by measuring the assay signal of replicates (*n* = 3) of the zero-concentration calibrator and determining the mean and standard deviation (SD) values. The limit of detection or assay sensitivity was then calculated as the concentration corresponding to the value of 3 standard deviations above the mean (mean +3SD).^65^

The working linear range was defined as a 20–80% inhibition rate (IC_20_–IC_80_).^66,67^

The statistical tests were performed with Prism 8.1.0 (GraphPad Software Inc., San Diego, CA, USA) after normality tests. The specific information on each test used can be found in the figure captions.

## Supplementary Material



## Data Availability

All data needed to evaluate the conclusions in the paper are present in the paper and/or the Supporting Information. Data from the trials in Mali were previously published in ref 33 and accessible on Clinical Epidemiology Database Resources (www.clinepidb.org) under study title “NECTAR4”. Other raw research data is available upon reasonable request from the corresponding authors.

## Notes

Additional nanoparticle characterization (Figures S1–S3), antibody physisorption optimization (Figures S4–S6), HRP2 assay development (Figure S7), matrix effect study (Figure S8), half-dipstick immunochromatographic assay (Figure S9), asexual parasite culture control data (Figure S10), estimation of LoD for gametocyte detection (Figure S11), clinical sensitivity and specificity calculations (Figures S12 and S13), and gametocyte density for samples used in clinical validation (Figure S14, reprinted (Adapted) with permission from ref 33. Copyright 2025, Elsevier, CC-BY) (PDF)

## ⋈

Department of Infection Biology, London School of Hygiene and Tropical Medicine, London, U.K
