# Supplementary material for: Evaluation of Malaria Parasite Transmission Competency Using a Nanozyme-Based Immunodiagnostic Targeting Female Gamete Antigen Release
Source: ACS Nano. 2025 Oct 6;19(41):36419–34. doi: 10.1021/acsnano.5c10298 (PMC12548336; doi:10.1021/acsnano.5c10298)
Supplement: Supplementary file 1 [file nn5c10298_si_001.pdf]

Supporting Information

Evaluation of Malaria Parasite Transmission Competency Using a Nanozyme-Based Immunodiagnostic Targeting Female Gamete Antigen Release

Tabasom Haghighi,[1,2] Adrian Najer,[1,3,] Marta Broto,[1] Farah A. Dahalan,[3] Alisje Churchyard,[3] Sabrina Yahiya,[3] Mufuliat T. Famodimu,[3] Mark Tunnicliff,[3] Aida Abdelwahed,[1] Mayumi Tachibana,[4] Tomoko Ishino,[5] Yaw Aniweh,[6] Gordon A. Awandare,[6] Almahamoudou Mahamar,[7] Leen N. Vanheer,[8] Teun Bousema,[9] Chris Drakeley,[8] Alassane Dicko,[7] William Stone,[8] Jake Baum,[3,10,]* and Molly M. Stevens[1,2,]**

[1]Department of Materials, Department of Bioengineering, and Institute of Biomedical Engineering, Imperial College London, London SW7 2AZ, UK.

[2]Kavli Institute for Nanoscience Discovery, Department of Physiology, Anatomy and Genetics, Department of Engineering Science, University of Oxford, OX1 3QU, Oxford, UK.

[3]Department of Life Sciences, Imperial College London, London, SW7 2AZ, UK.

[4]Division of Molecular Parasitology, Proteo-science Center, Ehime University, 454 Shitsukawa, Toon, Ehime 791-0295, Japan.

[5]Department of Parasitology and Tropical Medicine, Graduate School of Medical and Dental Sciences, Institute of Science Tokyo, 1-5-45, Yushima, Bunkyo-ku, Tokyo, 113-8519, Japan.

[6]West African Centre for Cell Biology of Infectious Pathogens (WACCBIP), College of Basic and Applied Sciences, University of Ghana, LG 54 Legon, Accra, Ghana.

[7]Clinical Research Unit of Bougouni-Ouelessebougou, Malaria Research and Training Centre, University of Sciences, Techniques, and Technologies of Bamako, Bamako BP 1805, Mali.

[8]Department of Infection Biology, London School of Hygiene and Tropical Medicine, London WC1E 7HT, UK.

[9]Radboud Institute for Health Sciences, Radboud University Medical Centre, Geert Grooteplein Zuid 28, 6525GA, Nijmegen, Netherlands.

[10]School of Biomedical Sciences, University of New South Wales, NSW 2052, Sydney, Australia.

Corresponding authors E-mails: a.najer@imperial.ac.uk; jake.baum@unsw.edu.au; molly.stevens@dpag.ox.ac.uk

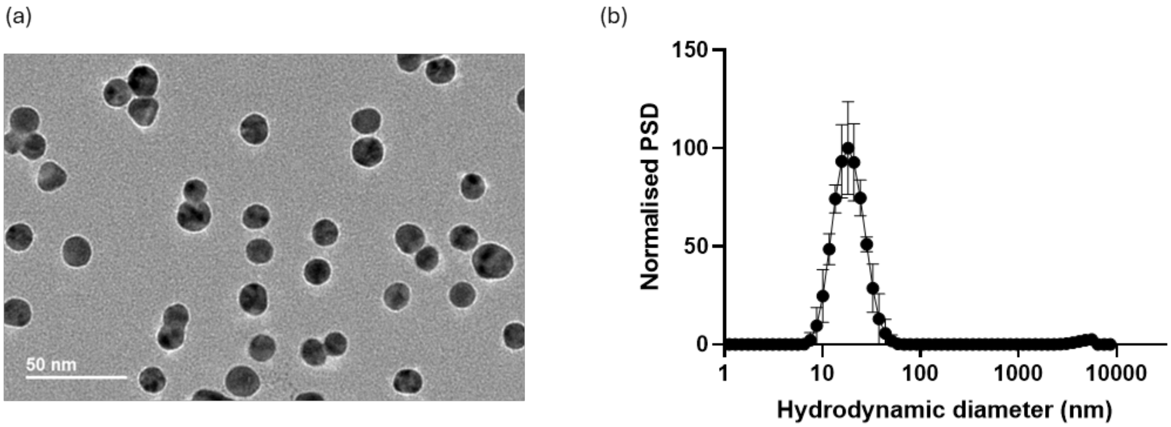

Figure S1. Characterization of Au seeds. (a) Transmission electron micrograph (TEM) of Au seeds. (b) Dynamic light scattering (DLS) characterization of Au seeds. Data represents mean ± s.d. (n = 3). PSD, particle size distribution. Normalized particle size distribution (intensity) is presented.

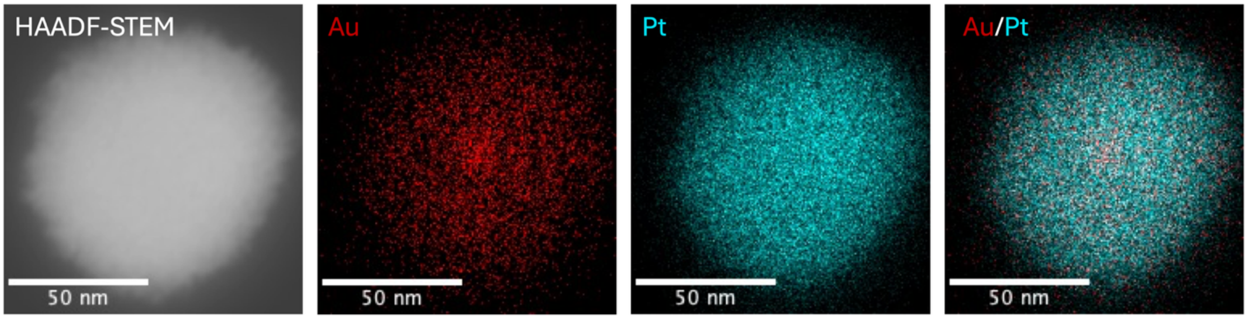

Figure S2. High-angle annular dark-field STEM (HAADF-STEM) image and energy dispersive X-ray (EDS) elemental mapping (Au, Pt, and merge). This is the full panel of main Figure 2d.

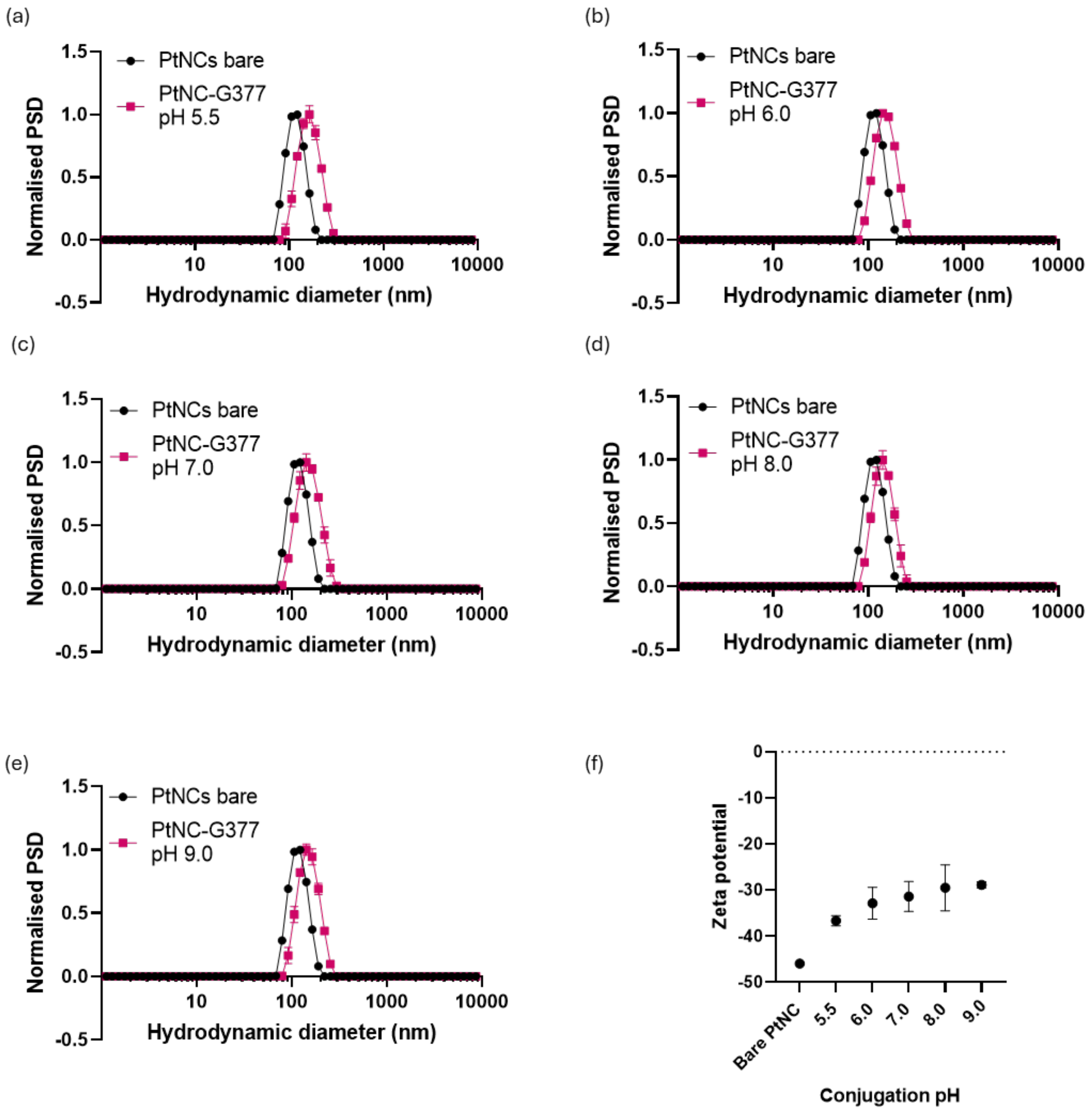

Figure S3. Dynamic light scattering (DLS) characterization of antibody-functionalized PtNCs. (a-e) DLS measurements showing normalized particle size distribution (intensity) for bare PtNCs (black, always the same reference particle shown) and anti-*Pf*G377 antibody-modified PtNCs (red) at different pH. Figure S3C was reproduced in this series from Figure 2e (in the main manuscript text) for comparison. (f) Zeta potential measurements of the particles conjugated with antibodies compared with bare PtNCs. Data represents mean ± s.d. (n = 3).

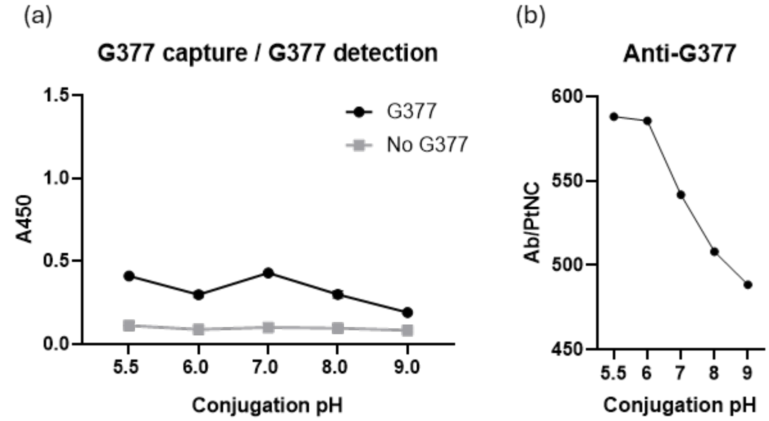

Figure S4. (a) The effect of conjugation pH on the performance of the antibody-modified PtNCs for the detection of the target antigen *Pf*G377. Graph of signal-to-noise ratio assessing the performance of the antibody-functionalized PtNCs in the selective detection of *Pf*G377. The black line represents a positive signal corresponding to the presence of antigen, while the gray line represents the negative control where no antigen was added to the assay (n = 2). (b) Number of antibodies immobilized onto PtNCs at different conjugation pHs for the anti-*Pf*G377 antibody system for the detection of *Pf*G377 antigen (n = *1*).

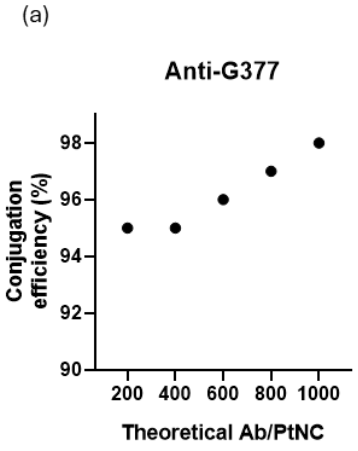

(b)

Antibody	Theoretical Ab/PtNC ratio	[Ab] added (µg/mL)	[Ab] in the supernatant (µg/mL)	Actual Ab/PtNC ratio
Anti- G377	200	15	0.796	189
	400	30	1.520	380
	600	45	1.673	577
	800	60	2.03	773
	1000	75	1.175	984

Figure S5. Antibody conjugation efficiency on PtNCs for (a) anti-*Pf*G377 antibodies (n = 1) and (b) the table summarizing the number of antibodies successfully immobilized on the surface of the PtNCs.

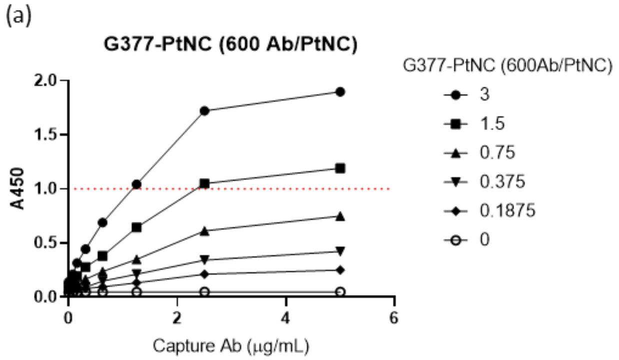

(b)

Target	G377
Capture Ab (μg/mL)	2
Detection Ab-PtNC (pM)	2 (600 Ab/PtNC)

Figure S6. Sandwich NLISA development and optimization using anti-*Pf*G377 antibody functionalized PtNCs. (a) Absorbance signal for the checkerboard titration assay for detecting *Pf*G377 (n = 1). The x-axis represents the different concentrations of the capture antibody. Each line on the graph corresponds to a different detection antibody-PtNC concentration (pM). (b) Optimized concentration of capture antibodies and Ab-PtNC detection units for detection of *Pf*G377.

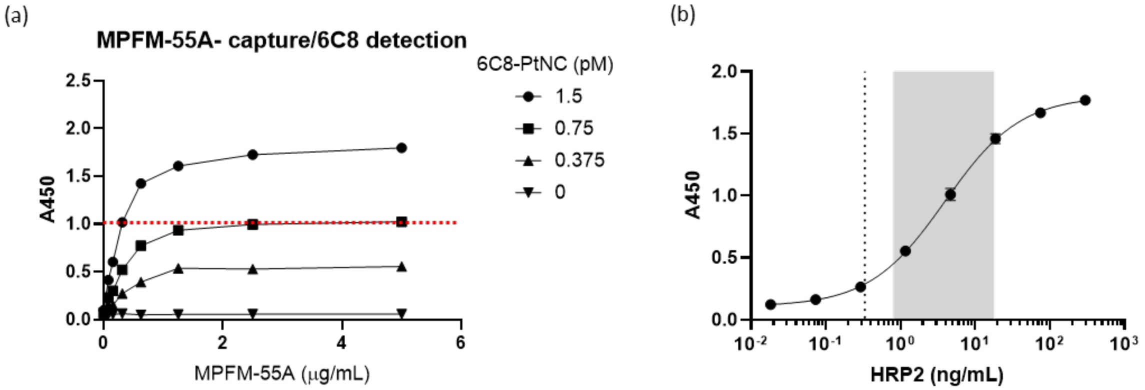

Figure S7. Sandwich NLISA development and optimization using anti-HRP2 antibody functionalized PtNCs. (a) Absorbance signal for the checkerboard titration assay for detecting HRP2 (n = 1). The x-axis represents the different concentrations of the capture antibody. Each line on the graph corresponds to a different detection antibody-PtNC concentration (pM). (b) Calibration curve of the HRP2 immunoassay using MPFM-55A/6C8-PtNC as capture/detection pair. The dashed line represents the limit of detection. The gray area represents the working range of the assay (n = 3). Mean +/- range.

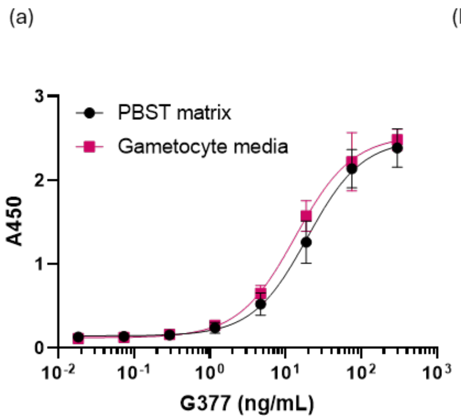

(b)

Parameters	Assay matrix	
	PBST	Gametocyte media
Min signal	0.14	0.12
Max signal	2.49	2.53
Hill slope	1.20	1.16
R^2	0.983	0.983
LOD (ng/mL)	**2.8**	**1.9**
Working range (ng/mL)	5.7 – 52	3.9 – 41

Figure S8. Matrix effect study on *Pf*G377-NLISA. (a) Serial dilution of *Pf*G377 was prepared in PBST and gametocyte culture medium. Data represents mean ± range (The data presented correspond to the average of two assays performed on two different days, $n = 3$ technical replicates each). (b) Sigmoidal regression curve parameters. The data were extracted from the four-parameter equation used to fit the standard curves in (a).

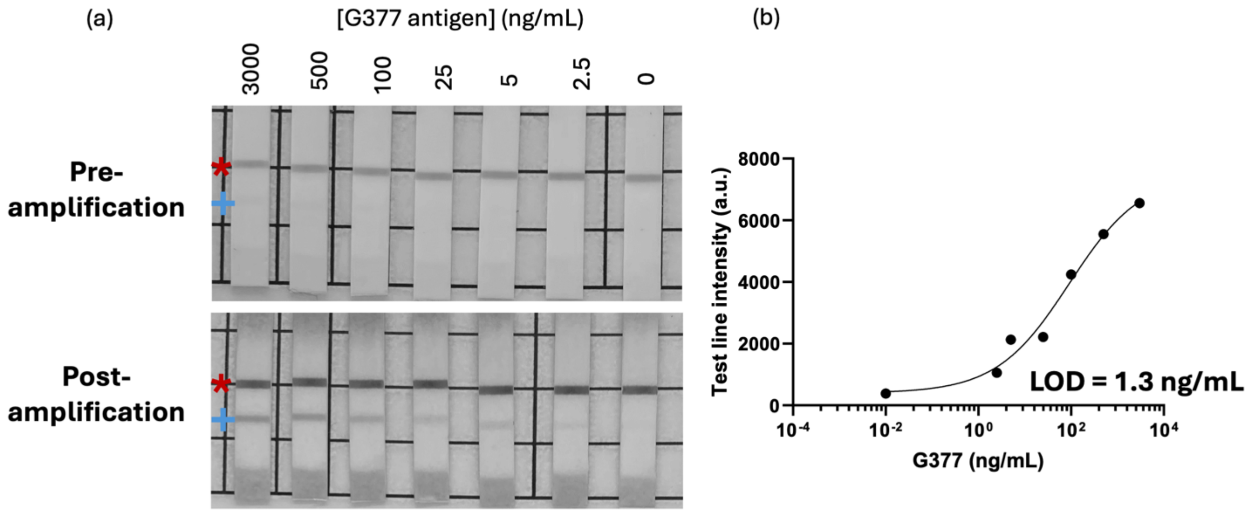

Figure S9. (a) Mobile phone images of *Pf*G377 serial dilution in FBS ran on a half-dipstick immunochromatographic assay using Ab-PtNC detection units at 400 Ab/PtNC ratio and 150 pM Ab-PtNC detection unit concentration before (top) and after (bottom) amplification in a mixture of CN/DAB and H_2O_2 for 5 min. The control lines, which consist of printed goat anti-rabbit IgG are highlighted with a red asterisk and the test lines, which consist of printed rabbit anti-*Pf*G377 polyclonal antibodies (the same as used on the PtNCs) are indicated with a blue plus sign. (b) Calibration curve of test line intensity obtained by mobile phone camera images from (a) post-amplification.

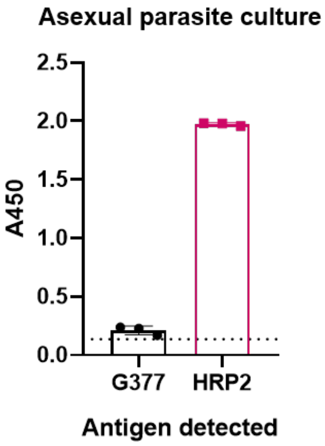

Figure S10. Assessment of the assay specificity. Asexual parasite culture samples were examined on the G377-NLISA and HRP2-NLISA platform and the amount of G377 and HRP2 protein present in the sample was measured. The dashed line represents the assay background noise of the gametocyte culture medium only in the absence of the antigens. Data is a combination of three independent biological replicates and *n* = 3 technical replicates each.

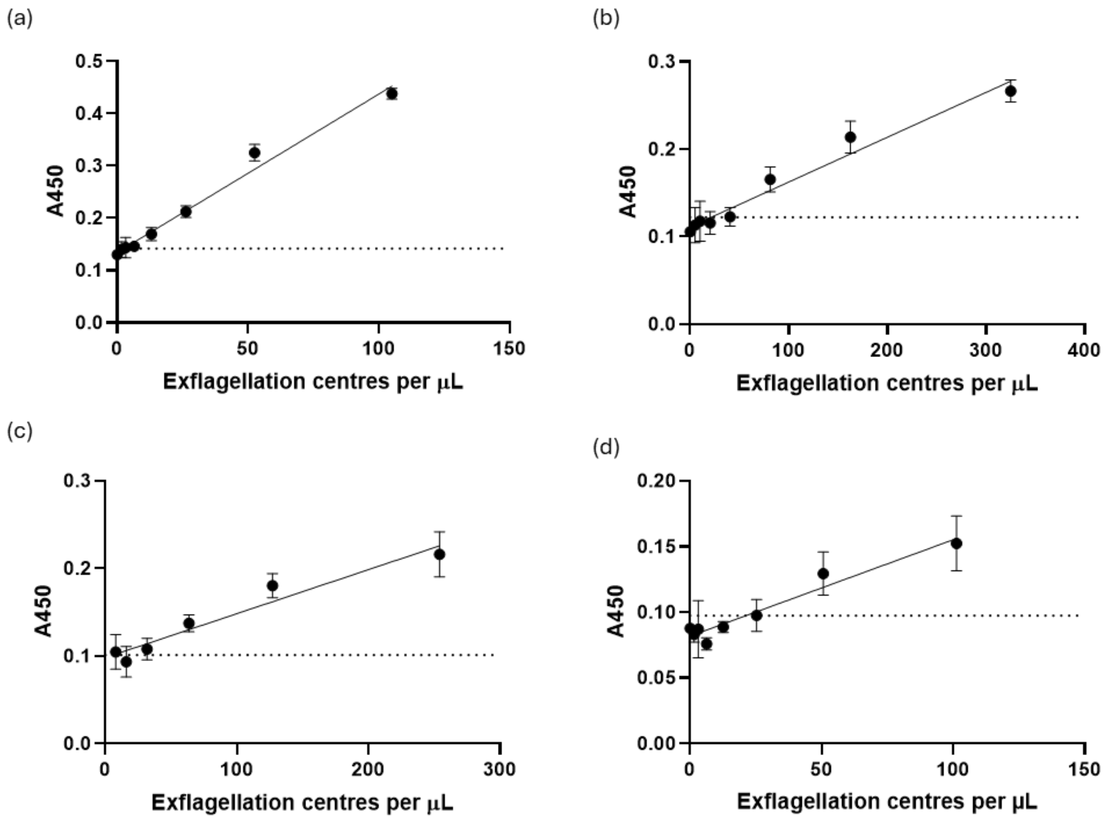

Biological Replicate	a	b	c	d
LoD in terms of Exflagellation centres/μL	2.49	21.53	5.93	21.23
Estimated LoD in terms of female gametocytes/μL	10.00	86.10	23.72	84.94

Figure S11. Assessment of the assay sensitivity through measuring the amount of *Pf*G377 released by NLISA from serial dilutions of gametocyte cell culture samples prepared in gametocyte media after activation. Graphs represent the linear regression plots of 4 independent biological replicates ($N = 4$, $n = 3$ technical repeats). The detection limit can be estimated as ~ 51 female gametocytes per μL of a sample (multiplied exflagellation centers, which refers to male gametocyte activation, by factor 4 to get female number).[1] Dotted horizontal lines represent mean background + 3 s.d.[2]

(a)

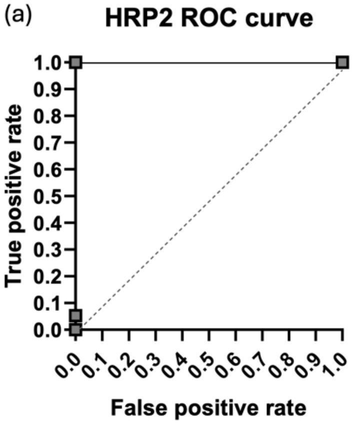

(b)

Cut-off (AU)	Sensitivity	Specificity	Youden Index
0.146	1	0.2	0.2
0.194	1	0.4	0.4
0.255	1	0.6	0.6
0.276	1	0.8	0.8
0.295	1	1	1
0.322	0.947	1	0.947
0.353	0.895	1	0.895
0.38	0.842	1	0.842
0.396	0.789	1	0.789
0.413	0.737	1	0.737
0.419	0.684	1	0.684
0.43	0.632	1	0.632
0.441	0.579	1	0.579

(c)

	Predicted Negative	Predicted Positive
True Negative (controls)	5	0
True Positive (malaria)	0	19

(d)

$$\text{Sensitivity} = \frac{19}{(19+0)} \times 100 = 100\%$$

$$\text{Specificity} = \frac{5}{(5+0)} \times 100 = 100\%$$

Figure S12. ROC curve analysis of the HRP2 malaria diagnostic assay (data from Figure 5b). (a) Receiver operating characteristic (ROC) curve showing sensitivity versus false positive rate. (b) Cut-off values with corresponding sensitivity, specificity, and Youden's Index, identifying the optimal threshold at 0.295 AU (sensitivity = 100%, specificity = 100%). (c) Confusion matrix summarizing classification outcomes for malaria-positive (*n* = 19) and malaria-negative (*n* = 5) samples at the optimal threshold. (d) Calculated sensitivity and specificity values. ROC analysis was performed using GraphPad Prism.

(a)

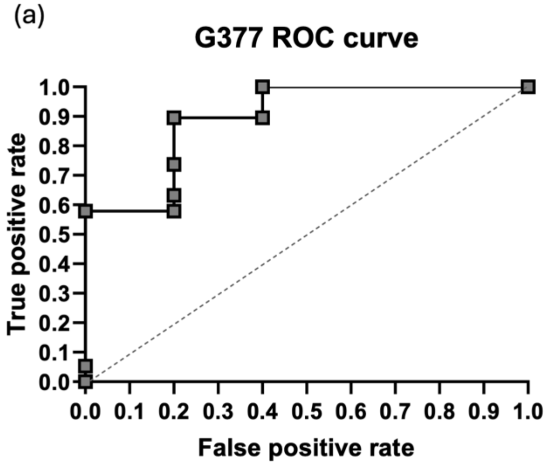

(b)

Cut-off (AU)	Sensitivity	Specificity	Youden Index
1.731	0	1	0
0.731	0.053	1	0.053
0.445	0.579	1	0.579
0.427	0.579	0.8	0.379
0.424	0.632	0.8	0.432
0.416	0.737	0.8	0.537
0.359	0.895	0.8	0.695
0.351	0.895	0.6	0.495
0.284	1	0.6	0.6
0.206	1	0	0

(c)

	Predicted Negative	Predicted Positive
True Negative (controls)	4	1
True Positive (malaria)	2	17

(d)

$$\text{Sensitivity} = \frac{17}{(17+2)} \times 100 = 89.5\%$$

$$\text{Specificity} = \frac{4}{(4+1)} \times 100 = 80.0\%$$

Figure S13. ROC curve analysis of the G377 malaria diagnostic assay (data from Figure 5c). (a) Receiver operating characteristic (ROC) curve showing sensitivity versus false positive rate. (b) Cut-off values with corresponding sensitivity, specificity, and Youden's Index, identifying the optimal threshold at 0.359 AU (sensitivity = 89.5%, specificity = 80.0%). (c) Confusion matrix summarizing classification outcomes for malaria-positive (n = 19) and malaria-negative (n = 5) samples at the optimal threshold. (d) Calculated sensitivity and specificity values. ROC curves were generated using standard ROC analysis in GraphPad Prism.

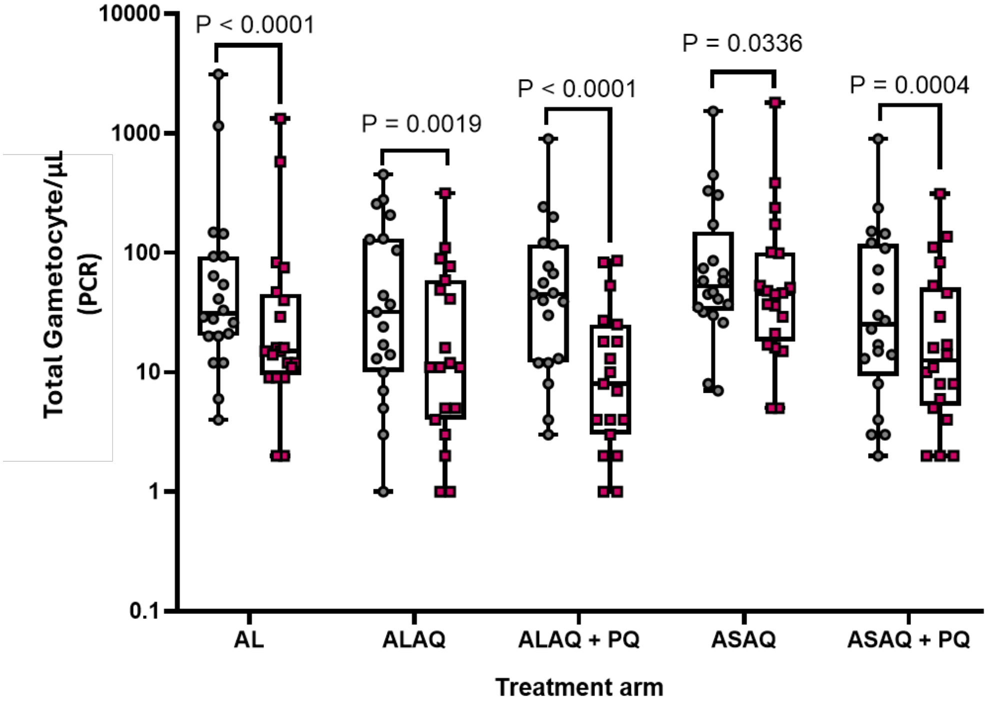

Figure S14. The effect of different artemisinin-based combination therapies (i.e., artemether–lumefantrine (AL) vs artemether–lumefantrine-amodiaquine (ALAQ) and artemether–lumefantrine-amodiaquine plus primaquine (+ PQ), artesunate-amodiaquine (ASAQ) vs artesunate-amodiaquine plus PQ) on the total blood circulating gametocyte density measured by RT-PCR at baseline (day 0, gray) and day 2 post-treatment (pink). Box and whisker plots represent median and quartiles. Wilcoxon paired test statistical analysis was performed. Reprinted (Adapted) with permission from reference[3]. Copyright 2025, Elsevier, CC-BY.

References

(1) Bousema, T.; Drakeley, C. Epidemiology and Infectivity of Plasmodium Falciparum and Plasmodium Vivax Gametocytes in Relation to Malaria Control and Elimination. *Clin Microbiol Rev* **2011**, *24* (2), 377–410. https://doi.org/10.1128/CMR.00051-10.

(2) Armbruster, D. A.; Pry, T. Limit of Blank, Limit of Detection and Limit of Quantitation. *Clin Biochem Rev* **2008**, *29 Suppl 1* (Suppl 1), S49-52.

(3) Mahamar, A.; Vanheer, L. N.; Smit, M. J.; Sanogo, K.; Sinaba, Y.; Niambele, S. M.; Diallo, M.; Dicko, O. M.; Diarra, R. S.; Maguiraga, S. O.; Youssouf, A.; Sacko, A.; Keita, S.; Samake, S.; Dembele, A.; Teelen, K.; Dicko, Y.; Traore, S. F.; Dondorp, A.; Drakeley, C.; Stone, W.; Dicko, A. Artemether–Lumefantrine–Amodiaquine or Artesunate–Amodiaquine Combined with Single Low-Dose Primaquine to Reduce Plasmodium Falciparum Malaria Transmission in Ouélessébougou, Mali: A Five-Arm, Phase 2, Single-Blind, Randomised Controlled Trial. *Lancet Microbe* **2025**, *6* (2), 100966. https://doi.org/10.1016/j.lanmic.2024.100966.
